# Diversity and pathogenicity of *Colletotrichum* species causing strawberry anthracnose in Taiwan and description of a new species, *Colletotrichum miaoliense* sp. nov.

**DOI:** 10.1038/s41598-020-70878-2

**Published:** 2020-09-04

**Authors:** Pei-Che Chung, Hung-Yi Wu, Yen-Wen Wang, Hiran A. Ariyawansa, Hsien-Pin Hu, Ting-Hsuan Hung, Shean-Shong Tzean, Chia-Lin Chung

**Affiliations:** 1 Miaoli District Agricultural Research and Extension Station, Council of Agriculture, Executive Yuan, Miaoli County, 36346 Taiwan; 2 Department of Plant Pathology and Microbiology, National Taiwan University, Taipei City, 10617 Taiwan

**Keywords:** Fungi, Pathogens, Microbiology

## Abstract

Strawberry is a small fruit crop with high economic value. Anthracnose caused by *Colletotrichum* spp. poses a serious threat to strawberry production, particularly in warm and humid climates, but knowledge of pathogen populations in tropical and subtropical regions is limited. To investigate the diversity of infectious agents causing strawberry anthracnose in Taiwan, a disease survey was conducted from 2010 to 2018, and *Colletotrichum* spp. were identified through morphological characterization and multilocus phylogenetic analysis with internal transcribed spacer, glyceraldehyde 3-phosphate dehydrogenase, chitin synthase, actin, beta-tubulin, calmodulin, and the intergenic region between *Apn2* and *MAT1-2-1* (ApMAT). Among 52 isolates collected from 24 farms/nurseries in Taiwan, a new species, *Colletotrichum miaoliense* sp. nov. (6% of all isolates), a species not previously known to be associated with strawberry, *Colletotrichum karstii* (6%), and three known species, *Colletotrichum siamense* (75%), *Colletotrichum fructicola* (11%), and *Colletotrichum boninense* (2%), were identified. The predominant species *C*. *siamense* and *C*. *fructicola* exhibited higher mycelial growth rates on potato dextrose agar and caused larger lesions on wounded and non-wounded detached strawberry leaves. *Colletotrichum boninense*, *C. karstii*, and *C. miaoliense* only caused lesions on wounded leaves. Understanding the composition and biology of the pathogen population will help in disease management and resistance breeding.

## Introduction

Strawberry (*Fragaria* × *ananassa* Duch.) is a popular small fruit crop with high economic and nutritive value. Strawberry is in high demand globally. From 2008 to 2018, the annual worldwide cultivation of strawberries increased from approximately 400 to 483 thousand hectares^1^. Although strawberries are native to temperate regions, they can also be grown in tropical and subtropical regions (sometimes under high-altitude conditions). The land areas devoted to strawberry cultivation in Colombia, Peru, Guatemala, Bolivia, and Taiwan in 2018 were 1,482 ha, 1,453 ha, 690 ha, 522 ha, and 506 ha, respectively^1^.

Anthracnose caused by *Colletotrichum* spp. is a serious threat to strawberry production, especially in warm and humid climates^2^. Rain-splashed conidia of *Colletotrichum* spp. serve as the major inoculum causing epidemics of strawberry anthracnose disease^3^. After landing on the plant surface, the conidia germinate, form appressoria, then penetrate the epidermal cells^4^. *Colletotrichum* spp. can infect various strawberry tissues, causing black spots or irregular spots on leaves, sunken black spots or necrosis lesions on petioles, stolons, and fruits, and wilting of the whole plant due to crown rot^2^. Under high humidity, concentric rings of acervuli with orange conidial masses can be observed on necrotic tissues. In the US state of Florida, anthracnose causes the death of up to 80% of seedlings in the nursery and yield losses of over 50% in the field^2^. In Taiwan, strawberry seedlings are propagated from March to September, and the high temperature, high humidity and heavy rainfall during this period provide a suitable environment for epidemics. From 2010 to 2016, anthracnose crown rot caused the loss of 30–40% of seedlings and ~ 20% of plants after transplanting^5^.

*Colletotrichum* spp. have traditionally been classified based on the shape of the conidia and appressorium, the presence of a seta or perithecium, and culture characteristics^6,7^. Using these criteria, early studies reported *C. acutatum*, *C. gloeosporioides*, and *C. fragariae* as strawberry anthracnose pathogens^2,8^. However, *Colletotrichum* spp. share similar features, and morphological characteristics can be influenced by environmental factors including culture media, light, and temperature^9−11^. Therefore, a polyphasic approach based on morphology and genetic characteristics was proposed for identification of *Colletotrichum* species^9^. A combination of multiple gene sequences, including internal transcribed spacer (ITS), glyceraldehyde 3-phosphate dehydrogenase (*GAPDH*), chitin synthase (*CHS-1*), actin (*ACT*), beta-tubulin (*TUB2*), calmodulin (*CAL*), and the intergenic region between *Apn2* and *MAT1-2-1* (ApMAT), can provide more molecular features to resolve different species in a *Colletotrichum* species complex^12,13^. Through multilocus sequence analysis coupled with morphological characterization, recent studies have identified many additional *Colletotrichum* species associated with strawberry, namely *C. acutatum*, *C. fioriniae*, *C. godetiae*, *C. nymphaeae*, *C. salicis* and *C. simmondsii* (*C. acutatum* species complex), *C. aenigma*, *C. changpingense*, *C. fructicola*, *C. gloeosporioides*, *C. siamense* and *C. theobromicola* (syn. *C. fragariae*) (*C. gloeosporioides* species complex) and *C. boninense* (*C. boninense* species complex)^6,12–18^.

Although strawberry is of great economic importance in Taiwan and anthracnose has become more destructive in the past decade, the pathogen population in Taiwan has not been systematically investigated. The causal agents of strawberry anthracnose were previously reported to be *C*. *gloeosporioides*^19^, *C. dematium*, *C*. *fragariae*, and *C*. *acutatum* (Plant Protection Information System; https://otserv2.tactri.gov.tw/ppm/), but information about the isolation, pathogenicity, morphology, and sequences of these species is not sufficient for species identification. Recently, based on analysis of multiple gene sequences, we identified *C*. *siamense* as the pathogen causing anthracnose crown rot^5^. To provide accurate information for disease control and resistance breeding, in this study we aimed to reveal the population composition of the infectious agents associated with strawberry anthracnose in Taiwan. Samples collected from the major strawberry-producing areas of Taiwan from 2010 to 2018 were subjected to morphological and multi-gene phylogenetic analyses. To further understand the in vitro and *in planta* aggressiveness of different *Colletotrichum* spp. at different temperatures, multiple representative isolates of each species were tested for mycelial growth rates in an artificial medium as well as the ability to cause lesions on wounded or non-wounded strawberry leaves. Since population analysis of *Colletotrichum* spp. causing strawberry anthracnose has only been reported for species from the UK^14^ and China [Anhui, Hainan (only one isolate), Hebei, Hubei, Liaoning, Shandong, and Zhejiang Provinces and Beijing and Shanghai cities]^16–18^, which, with the exception of Hainan, are geographical regions located at higher latitudes (30–53°N) relative to Taiwan (24.5°N), this study will provide insights into the biology of strawberry anthracnose disease in subtropical regions.

## Results

### Molecular identification and phylogenetic analysis

*Colletotrichum* spp. isolates were first identified at the species complex level. Among 52 *Colletotrichum* spp. isolates sampled from the major strawberry-producing areas of Taiwan, 45 (86.5%) isolates belonged to the *C*. *gloeosporioides* species complex, 4 (7.7%) belonged to the *C*. *boninense* species complex, and 3 (5.8%) belonged to the *C*. *acutatum* species complex (Table 1).

**Table 1:** List of *Colletotrichum* spp. associated with strawberry anthracnose in Taiwan.

Species		Isolate^a^	Strawberry tissue	Collection date	Sampling site	GenBank accession number^b^						
						ITS	*GAPDH*	*CHS1*	*ACT*	*TUB2*	*CAL*	ApMAT
***C. acutatum*** **species complex**												
	*C. miaoliense* sp. nov	**ML1040**	crown	2016/10/28	Shitan Township, Miaoli County	MK908419	MK908470	MK908522	MK908573	MK908624	-^c^	-
		**ML1042**	leaf	2016/11/24	Renai Township, Nantou County	MK908420	MK908471	MK908523	MK908574	MK908625	-	-
		**ML1794**	leaf	2018/07/04	Renai Township, Nantou County	MK908421	MK908472	MK908524	MK908575	MK908626	-	-
***C. boninense*** **species complex**												
	*C. boninense*	**ML521**	leaf	2013/01/21	Taian Township, Miaoli County	MK908424	MK908475	MK908527	MK908578	MK908629	MK908677	-
	*C. karstii*	**ML351**	leaf	2012/07/06	Shitan Township, Miaoli County	MK908422	MK908473	MK908525	MK908576	MK908627	MK908675	-
		**ML442**	leaf	2012/07/04	Nanchuang Township, Miaoli County	MK908423	MK908474	MK908526	MK908577	MK908628	MK908676	-
		**ML1792**	leaf	2018/05/16	Renai Township, Nantou County	MK908425	MK908476	MK908528	MK908579	MK908630	MK908678	-
***C. gloeosporioides*** **species complex**												
	*C. fructicola*	**ML348**	leaf	2012/07/06	Shitan Township, Miaoli County	MK908461	MK908513	MK908564	MK908615	MK908666	MK908714	MK908758
		ML353	root	2012/07/10	Shitan Township, Miaoli County	MK908462	MK908514	MK908565	MK908616	MK908667	MK908715	MK908759
		**ML356**	crown	2012/07/10	Shitan Township, Miaoli County	MK908463	MK908515	MK908566	MK908617	MK908668	MK908716	MK908760
		**ML368**	stolon	2012/07/24	Dahu Township, Miaoli County	MK908464	MK908516	MK908567	MK908618	MK908669	MK908717	MK908761
		ML818	crown	2016/07/06	Renai Township, Nantou County	MK908468	MK908520	MK908571	MK908622	MK908673	MK908721	MK908765
		ML1012	leaf	2016/11/09	Renai Township, Nantou County	MK908469	MK908521	MK908572	MK908623	MK908674	MK908722	MK908766
	*C. siamense*	ML040	fruit	2010/03/22	Gongguan Township, Miaoli County	MK908426	MK908477	MK908529	MK908580	MK908631	MK908679	MK908723
		ML041	fruit	2010/03/24	Dahu Township, Miaoli County	MK908427	MK908478	MK908530	MK908581	MK908632	MK908680	MK908724
		ML048	fruit	2010/05/12	Dahu Township, Miaoli County	MK908428	MK908479	MK908531	MK908582	MK908633	MK908681	MK908725
		ML076	stolon	2010/08/27	Dahu Township, Miaoli County	MK908429	MK908480	MK908532	MK908583	MK908634	MK908682	MK908726
		**ML133**	crown	2011/10/28	Dahu Township, Miaoli County	MK174223	MK908481	MK174224	MK174225	MK174226	MK174227	MK174228
		ML177	fruit	2012/03/09	Dahu Township, Miaoli County	MK908430	MK908482	MK908533	MK908584	MK908635	MK908683	MK908727
		ML275	crown	2012/06/04	Dahu Township, Miaoli County	MK908431	MK908483	MK908534	MK908585	MK908636	MK908684	MK908728
		ML284	crown	2012/06/04	Shitan Township, Miaoli County	MK908432	MK908484	MK908535	MK908586	MK908637	MK908685	MK908729
		ML293	root	2012/4/25	Guanxi Township, Hsinchu County	MK908433	MK908485	MK908536	MK908587	MK908638	MK908686	MK908730
		ML294	crown	2012/06/13	Dahu Township, Miaoli County	MK908434	MK908486	MK908537	MK908588	MK908639	MK908687	MK908731
		ML296	crown	2012/06/13	Dahu Township, Miaoli County	MK908435	MK908487	MK908538	MK908589	MK908640	MK908688	MK908732
		ML320	crown	2012/06/27	Taian Township, Miaoli County	MK908436	MK908488	MK908539	MK908590	MK908641	MK908689	MK908733
		ML328	crown	2012/07/02	Shitan Township, Miaoli County	MK908437	MK908489	MK908540	MK908591	MK908642	MK908690	MK908734
		ML372	crown	2012/07/26	Shitan Township, Miaoli County	MK908438	MK908490	MK908541	MK908592	MK908643	MK908691	MK908735
		ML393	stolon	2012/07/26	Shitan Township, Miaoli County	MK908439	MK908491	MK908542	MK908593	MK908644	MK908692	MK908736
		ML416	crown	2012/08/15	Dahu Township, Miaoli County	MK908440	MK908492	MK908543	MK908594	MK908645	MK908693	MK908737
		ML418	crown	2012/08/15	Dahu Township, Miaoli County	MK908441	MK908493	MK908544	MK908595	MK908646	MK908694	MK908738
		ML419	root	2012/08/06	Shitan Township, Miaoli County	MK908442	MK908494	MK908545	MK908596	MK908647	MK908695	MK908739
		ML443	crown	2012/07/26	Shitan Township, Miaoli County	MK908443	MK908495	MK908546	MK908597	MK908648	MK908696	MK908740
		ML458	crown	2012/08/15	Dahu Township, Miaoli County	MK908444	MK908496	MK908547	MK908598	MK908649	MK908697	MK908741
		ML461	leaf	2012/09/12	Gongguan Township, Miaoli County	MK908445	MK908497	MK908548	MK908599	MK908650	MK908698	MK908742
		ML462	crown	2012/09/12	Gongguan Township, Miaoli County	MK908446	MK908498	MK908549	MK908600	MK908651	MK908699	MK908743
		ML463	root	2012/09/12	Gongguan Township, Miaoli County	MK908447	MK908499	MK908550	MK908601	MK908652	MK908700	MK908744
		ML464	crown	2012/09/13	Shitan Township, Miaoli County	MK908448	MK908500	MK908551	MK908602	MK908653	MK908701	MK908745
		ML469	crown	2012/09/18	Dahu Township, Miaoli County	MK908449	MK908501	MK908552	MK908603	MK908654	MK908702	MK908746
		ML471	crown	2012/09/18	Dahu Township, Miaoli County	MK908450	MK908502	MK908553	MK908604	MK908655	MK908703	MK908747
		ML476	root	2012/09/18	Dahu Township, Miaoli County	MK908451	MK908503	MK908554	MK908605	MK908656	MK908704	MK908748
		ML477	crown	2012/09/18	Dahu Township, Miaoli County	MK908452	MK908504	MK908555	MK908606	MK908657	MK908705	MK908749
		ML485	crown	2012/10/04	Guoxing Township, Nantou County	MK908453	MK908505	MK908556	MK908607	MK908658	MK908706	MK908750
		ML490	crown	2012/11/13	Gongguan Township, Miaoli County	MK908454	MK908506	MK908557	MK908608	MK908659	MK908707	MK908751
		ML491	crown	2012/11/13	Gongguan Township, Miaoli County	MK908455	MK908507	MK908558	MK908609	MK908660	MK908708	MK908752
		ML494	crown	2012/12/10	Dahu Township, Miaoli County	MK908456	MK908508	MK908559	MK908610	MK908661	MK908709	MK908753
		ML513	crown	2013/01/11	Shitan Township, Miaoli County	MK908457	MK908509	MK908560	MK908611	MK908662	MK908710	MK908754
		**ML540**	stolon	2013/04/22	Gongguan Township, Miaoli County	MK908458	MK908510	MK908561	MK908612	MK908663	MK908711	MK908755
		ML608	stolon	2013/07/10	Shitan Township, Miaoli County	MK908459	MK908511	MK908562	MK908613	MK908664	MK908712	MK908756
		**ML612**	leaf	2013/07/10	Shitan Township, Miaoli County	MK908460	MK908512	MK908563	MK908614	MK908665	MK908713	MK908757
		ML617	crown	2013/07/18	Dahu Township, Miaoli County	MK908465	MK908517	MK908568	MK908619	MK908670	MK908718	MK908762
		ML754	leaf	2016/03/17	Chiayi County	MK908466	MK908518	MK908569	MK908620	MK908671	MK908719	MK908763
		ML762	fruit	2016/03/28	Dahu Township, Miaoli County	MK908467	MK908519	MK908570	MK908621	MK908672	MK908720	MK908764

^a^Isolates in bold are representatives of each *Colletotrichum* spp. selected for mycelial growth and pathogenicity assays.

^b^ITS: internal transcribed spacer; *GAPDH*: glyceraldehyde 3-phosphate dehydrogenase; *CHS-1*: chitin synthase; *ACT*: actin; *TUB2*: beta-tubulin; *CAL*: calmodulin; ApMAT: intergenic sequence between *Apn2* DNA lyase and *MAT1-2-1.*

^c^-: not available.

**Figure 1:**
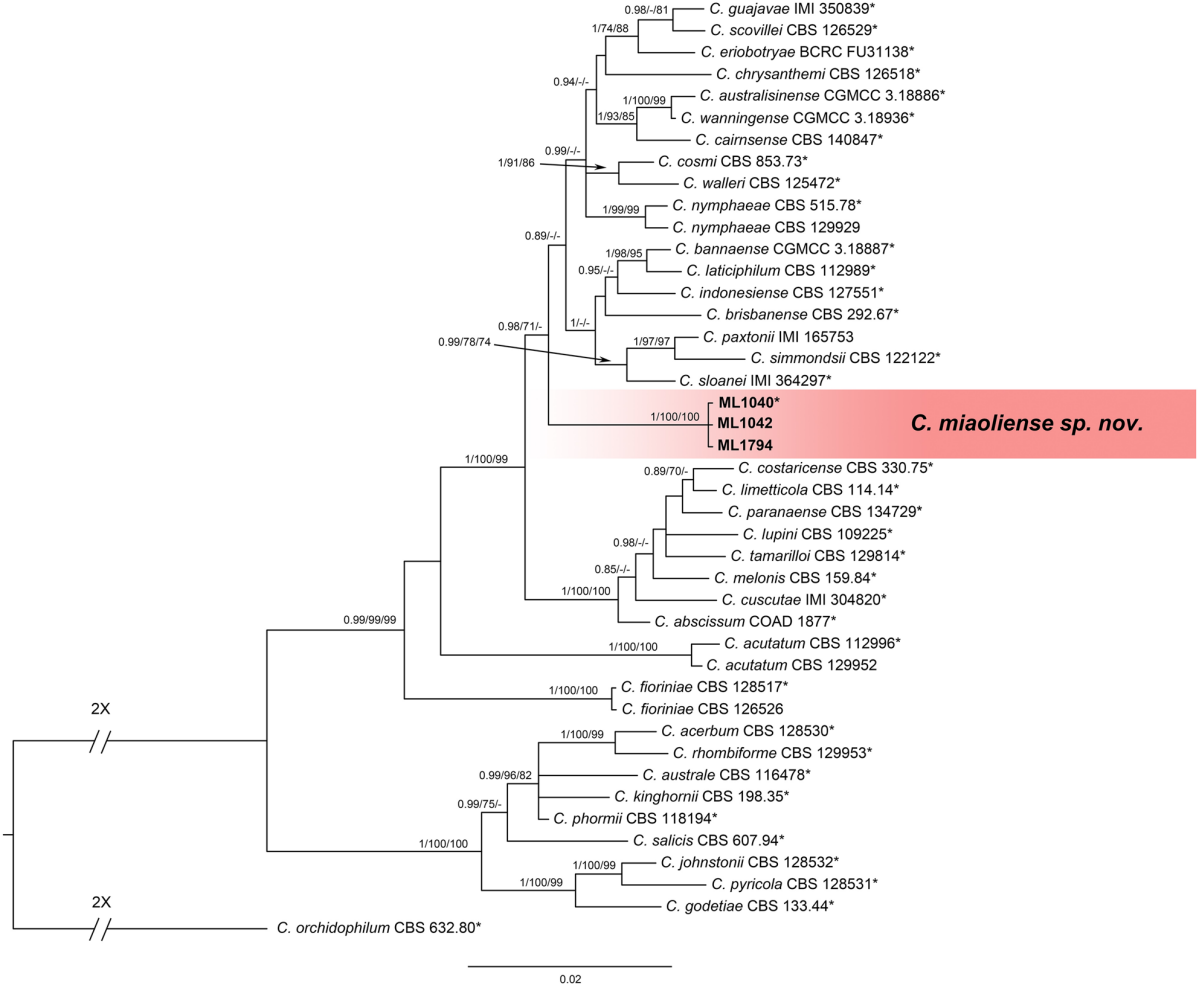
A Bayesian inference phylogenetic tree of the *C. acutatum* species complex. The phylogenetic tree was built using concatenated sequences of the ITS and the *GAPDH*, *ACT*, *CHS-1* and *TUB2* genes. Bayesian inference (BI) posterior values above 0.9 and bootstrap support values from maximum likelihood (ML) and maximum parsimony (MP) above 70% are shown at each node (BI/ML/MP). *C. orchidophilum* CBS 632.80 was used as the outgroup. *Indicates the ex-type strains. Strains isolated in this study are shown in bold.

To further analyze the *C*. *acutatum* species complex, 3 isolates together with 40 reference isolates, including the outgroup *C*. *orchidophilum* (CBS 632.80), were used to construct phylogenetic trees with five gene sequences (ITS, *GAPDH*, *CHS-1*, *ACT*, and *TUB2*) (Table 1 and Supplementary Table S1) following Damm et al.^12^ and Fu et al.^20^. The final data matrix contained a total of 1,821 characters with gaps (ITS: 1–540, *GAPDH*: 541–799, *CHS-1*: 800–1,081, *ACT*: 1,082–1,329, *TUB2*: 1,330–1,821), of which 237 characters were parsimony informative, 174 parsimony uninformative, and 1,410 constant. After 2,000,000 generations of topological convergence via Bayesian inference (BI) analysis, 2,378 trees were obtained. The first 25% of the trees were discarded, representing the burn-in phase of the analyses, and the remaining trees were used to calculate the Bayesian posterior probabilities in the majority rule consensus tree (Fig. 1). The maximum likelihood (ML) analysis resulted in a best scoring RAxML tree with a final optimized likelihood value of − 6,726.174303. The most parsimonious tree resulted from the maximum parsimony (MP) analysis received tree length = 692, consistency index (CI) = 0.714, and retention index (RI) = 0.843. All three isolates (ML1040, ML1042, ML1794) were grouped in a distinct clade with significant statistical support in the multilocus phylogenetic analysis (1/100/100, BI/ML/MP) (Fig. 1) and the single gene trees of *GAPDH*, *CHS-1*, and *TUB2* (Supplementary Fig. S1). This clade was distinct from all other known species, and is herein described as a new species, *C. miaoliense* sp. nov.

**Figure 2:**
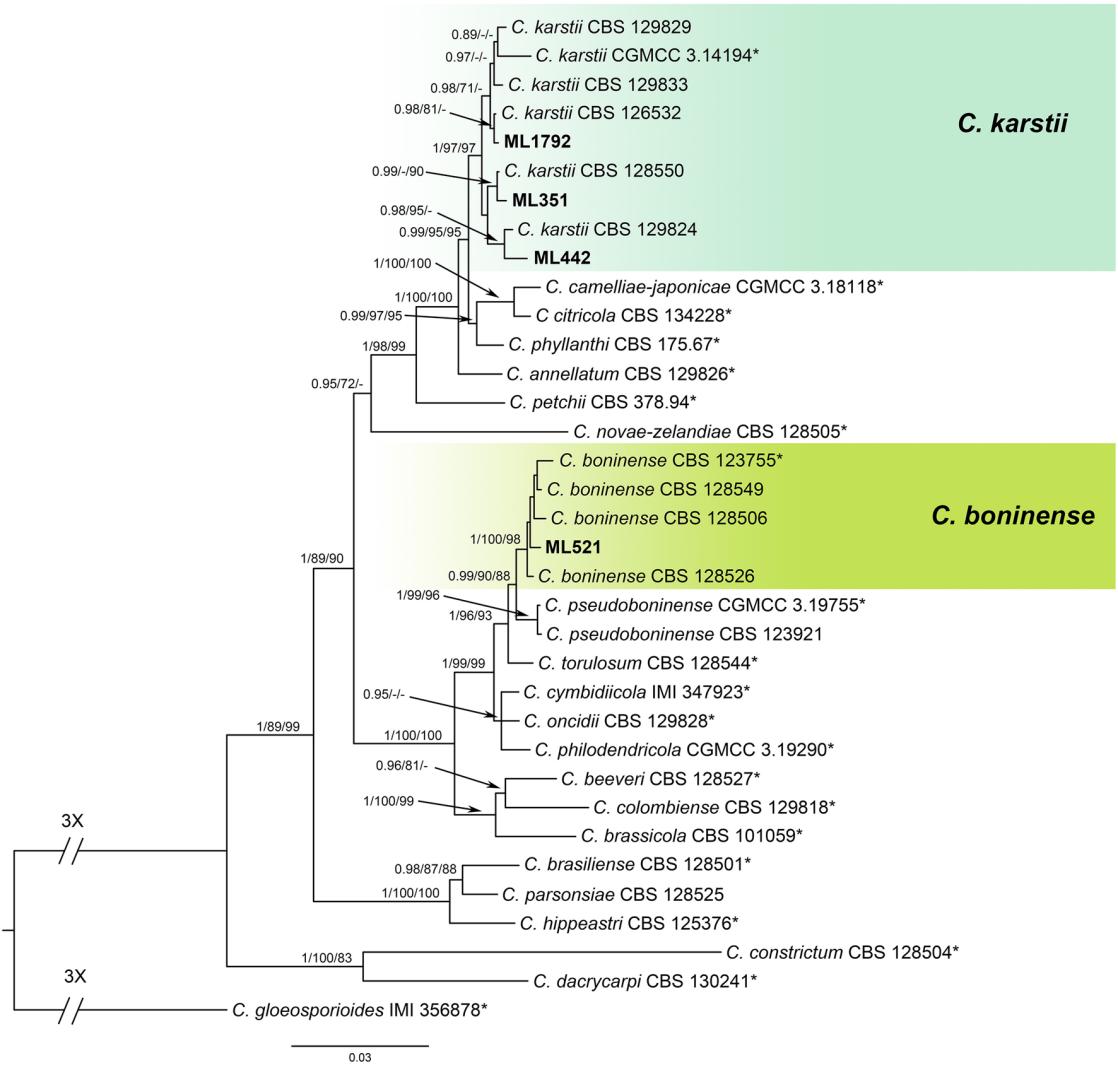
A Bayesian inference phylogenetic tree of the *C. boninense* species complex. A phylogram was built using concatenated sequences of the ITS and the *GAPDH*, *ACT*, *CHS-1*, *TUB2* and *CAL* genes. Bayesian inference (BI) posterior values above 0.9 and bootstrap support values from maximum likelihood (ML) and maximum parsimony (MP) above 70% are shown at each node (BI/ML/MP). *C. gloeosporioides* IMI 356878 was used as the outgroup. *Indicates the ex-type strains. Strains isolated in this study are shown in bold.

To analyze the phylogeny of the *C*. *boninense* species complex, six gene sequences (ITS, *GAPDH*, *CHS-1*, *ACT*, *TUB2*, and *CAL*) from 4 isolates together with 31 reference isolates, including the outgroup sequence of *C*. *gloeosporioides* (IMI 356878), were used to construct phylogenetic trees (Table 1 and Supplementary Table S1). The final data matrix contained a total of 2,363 characters with gaps (ITS: 1–558, *GAPDH*: 559–852, *CHS-1*: 853–1,132, *ACT*: 1,133–1,411, *TUB2*: 1,412–1,914, *CAL*: 1,915–2,363), of which 365 characters were parsimony informative, 407 parsimony uninformative, and 1,591 constant. After 1,187,000 generations of topological convergence via BI analysis, 492 trees were obtained. The first 25% of the trees were discarded, representing the burn-in phase of the analyses, and the remaining trees were used to calculate the Bayesian posterior probabilities in the majority rule consensus tree (Fig. 2). The ML analysis resulted in a best scoring RAxML tree with a final optimized likelihood value of − 10,025.941645. The most parsimonious tree resulted from the MP analysis received tree length = 1,281, CI = 0.774, and RI = 0.839. In single gene trees of *GAPDH*, *CHS-1*, and *TUB2* and the multilocus phylogenetic tree, three isolates (ML351, 442, 1792) clustered with strong statistical support in the clade containing the type strain CGMCC 3.14194 and other related isolates of *C. karstii* (Fig. 2; single gene trees not shown). In single gene trees of *GAPDH* and *CAL* and the multilocus phylogenetic tree, the isolate ML521 clustered with strong statistical support in the clade containing the type strain CBS 123755 and other related isolates of *C*. *boninense* (Fig. 2; single gene trees not shown).

**Figure 3:**
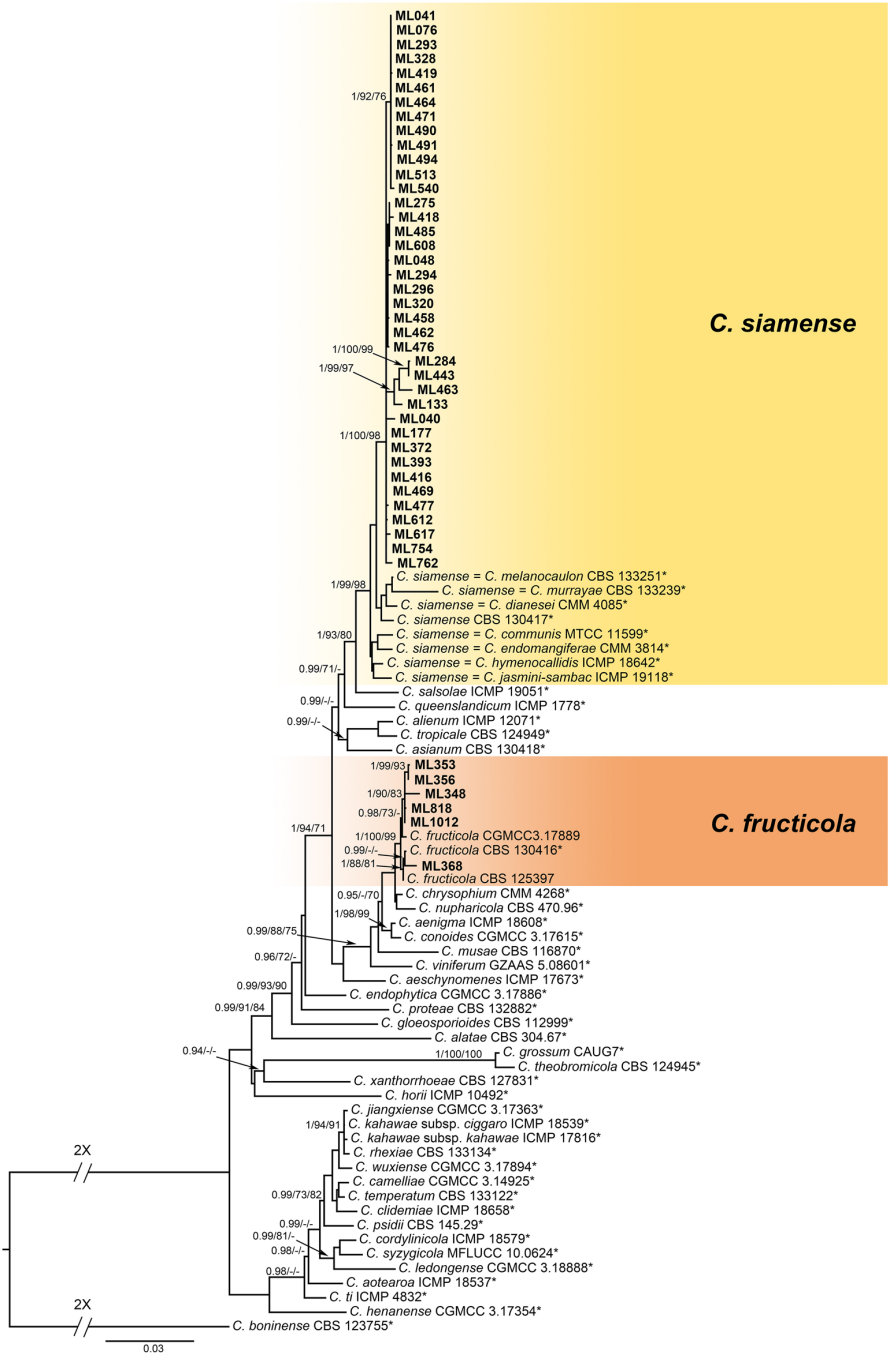
A Bayesian inference phylogenetic tree of the *C. gloeosporioides* species complex. The phylogenetic tree was built using concatenated sequences of the ITS, ApMAT, and the *GAPDH*, *ACT*, *CHS-1*, *TUB2*, *CAL* genes. Bayesian inference (BI) posterior values above 0.9 and bootstrap support values from maximum likelihood (ML) and maximum parsimony (MP) above 70% are shown at each node (BI/ML/MP). *C*. *boninense* CBS 123755 was used as the outgroup. *Indicates the ex-type strains. Strains isolated in this study are shown in bold.

To identify species in the *C*. *gloeosporioides* species complex, a combination of seven gene sequences (ITS, *GAPDH*, *CHS-1*, *ACT*, *TUB2*, *CAL* and ApMAT) from 45 isolates together with 47 reference isolates, including the outgroup sequence of *C*. *boninense* (CBS 123755), were used to construct phylogenetic trees (Table 1 and Supplementary Table S1). The final data matrix contained a total of 3,571 characters with gaps (ITS: 1–553, *GAPDH*: 554–808, *CHS-1*: 809–1,051, *ACT*: 1,052–1,325, *TUB2*: 1,326–2,028, *CAL*: 2,029–2,728, ApMAT: 2,729–3,571), of which 655 characters were parsimony informative, 735 parsimony uninformative, and 2,181 constant. After 6,574,000 generations of topological convergence via BI analysis, 9,864 trees were obtained. The first 25% of the trees were discarded, representing the burn-in phase of the analyses, and the remaining trees were used to calculate the Bayesian posterior probabilities in the majority rule consensus tree (Fig. 3). The ML analysis resulted in a best scoring RAxML tree with a final optimized likelihood value of − 18,514.217014. The most parsimonious tree resulted from the MP analysis received tree length = 2,400, CI = 0.722, and RI = 0.876. In single gene trees of *TUB2*, *CAL*, and ApMAT and the multilocus phylogenetic tree, 39 isolates clustered with strong statistical support in the clade containing the type strain CBS 130417 and other related isolates of *C. siamense* (Fig. 3; single gene trees not shown). The 39 isolates formed a subclade with a high support value (1/100/98, BI/ML/MP) (Fig. 3). In single gene trees of *GAPDH*, *ACT*, *TUB2*, *CAL*, and ApMAT and the multilocus phylogenetic tree, six isolates clustered with strong statistical support in the clade containing the type strain CBS 130416 and other related isolates of *C*. *fructicola* (Fig. 3; single gene trees not shown).

## Taxonomy

**Figure 4:**
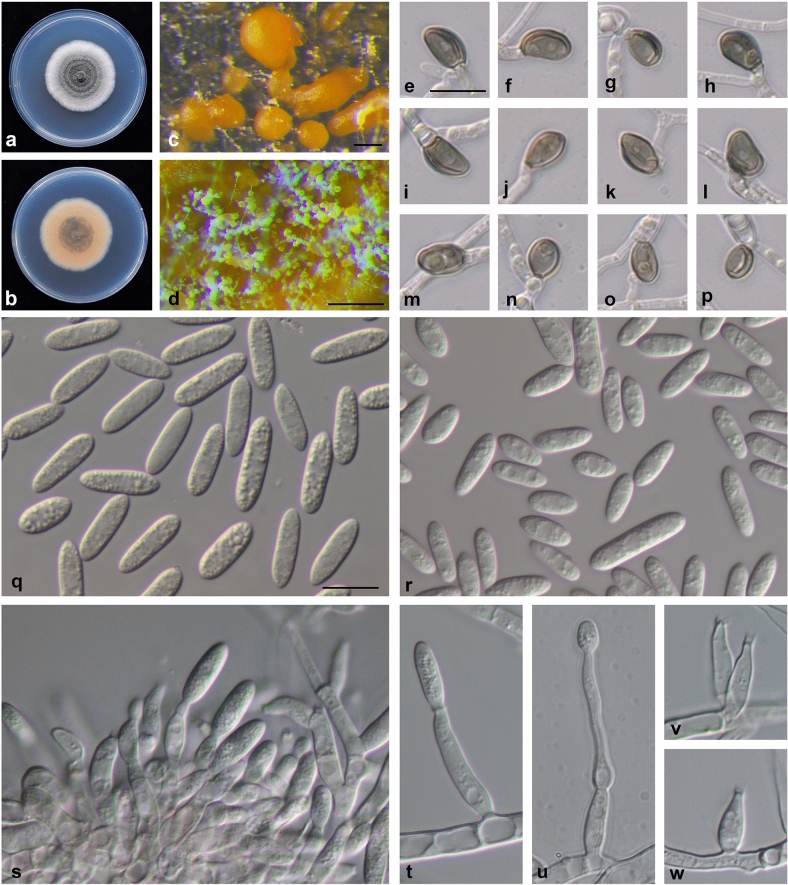
*Colletotrichum miaoliense* sp. nov. ML1040. (**a**) Upper side of colony; (**b**) reverse side of colony; (**c**, **d**) conidiomata; (**e**–**p**) appressorium (induced in dH_2_O on a microscope slide); (**q**) conidia from conidiomata; (**r**) conidia from aerial mycelium; (**s**–**w**) conidiophores. (**a**, **b**) on potato dextrose agar (PDA); (**c**, **d**) and (**q**–**w**) on 1/4-strength PDA. Scale bars: (**c**, **d**) = 0.2 mm, (**e**) = 10 μm, applies to (**f**–**p**, **q**) = 10 μm, applies to (**r**–**w**).

Based on morphological traits and multilocus phylogenetic analysis, the 52 isolates were assigned to five *Colletotrichum* spp. including one new taxon (*C*. *miaoliense* sp. nov.) (Fig. 4; described in detail below), one newly recorded taxon in strawberry (*C*. *karstii*), and three species known to be associated with strawberry anthracnose (*C. boninense*, *C. fructicola* and *C. siamense*) (Supplementary Fig. S2–S5). The colony features that developed at 25 °C on PDA and 1/4 PDA were all white to grey, with orange conidia ooze. *C*. *siamense* ML133 and *C. karstii* ML351 produced abundant conidia when cultured on 1/4 PDA at 25 °C; *C*. *boninense* ML521 produced more conidia on PDA at 25 °C; *C. fructicola* ML348 and *C. miaoliense* ML1040 sporulated more abundantly on 1/4 PDA at 30 °C. The conidium and appressorium measurements of the five *Colletotrichum* spp. (isolates from this study and the type strains) are listed in Supplementary Table S2. The conidia produced by *C. miaoliense* ML1040 were longer [length to width (L/W) ratio = 3.4] (Fig. 4) than the conidia of the other four species in this study (L/W ratio = 2.3–3) (Fig. S2–S5; Supplementary Table S2).

### Colletotrichum miaoliense sp. nov. P. C. Chung & H. Y. Wu. Figure 4

#### MycoBank number MB835424

Etymology: The epithet *miaoliense* specifically refers to Miaoli County, Taiwan, where the new taxon was discovered.

*Sexual morph* not observed. *Asexual morph* observed on 1/4 PDA [BCRC FU31304 (= NTUCC 20*-*001-1, ML1040)]. Vegetative hyphae 3–6 µm in diameter, hyaline, smooth-walled, septate, branched. Chlamydospores not observed. Sporodochia developed, conidiophores formed directly on hyphae. Conidiophores hyaline, smooth-walled, simple or branched. Conidiogenous cells hyaline, smooth-walled, cylindrical to ampulliform, often integrated, occasionally polyphialidic; phialides discrete, 5.9–26.4 µm (x̅ = 13.3 ± 4.8, n = 55) in length, apical opening 1.1–2.6 µm in diameter (1.7 ± 0.3, n = 55). Conidia hyaline, smooth-walled, aseptate, straight, fusiform to cylindrical, acute ends, 11.2–17 × 3.3–5 µm (x̅ = 14.2 ± 1.1 × 4.1 ± 0.3 µm, n = 100), L/W ratio = 3.4. Conidia from aerial hyphae varied in size (6.6–20 × 2.9–4.9 µm, x̅ = 11.2 ± 2.5 × 3.8 ± 0.4 µm, n = 100), L/W ratio = 3.0. Seta absent. Appressoria single or in loose clusters, pale brown, smooth-walled, elliptical to clavate, entire edge, 5.9–9.1 × 4–6.0 µm (x̅ = 7.5 ± 1.1 × 5 ± 0.6 µm, n = 27), L/W ratio = 1.5.

Culture features: Colonies on PDA flat to somewhat raised, margin entire; mycelium partly floccose, white to pale olivaceous grey; sporodochia orange, scattered in rings, reverse bright orange to orange; average 4.2 cm in diameter in 7 days at 25 °C. Conidia ooze was visible as an orange mass.

Material examined: Taiwan, Miaoli County, Shitan Township, from crown rot of *Fragaria* × *ananassa*, 28 Oct. 2016, P.-C. Chung; holotype NTUH 20*-*001-1, ex-holotype living culture BCRC FU31304 (= NTUCC 20*-*001-1, ML1040).

Additional materials examined: Taiwan, Nantou County, Renai Township, from leaf spot of *Fragaria* × *ananassa*, 24 Nov. 2016, P.-C. Chung, NTUH 20*-*001-2; living culture NTUCC 20*-*001-2 (= ML1042). Taiwan, Nantou County, Renai Township, from leaf spot of *Fragaria* × *ananassa*, 4 Jul. 2018, P.-C. Chung; NTUH 20*-*001-3, living culture NTUCC 20*-*001-3 (= ML1794). Known distribution: Miaoli and Nantou Counties, Taiwan.

*Notes* Three isolates of *C. miaoliense* were collected from Miaoli County and Nantou County, Taiwan. Multilocus analysis indicated that *C. miaoliense* forms a robust clade clearly distinct from all the other known species in the *C. acutatum* species complex. Of the six *Colletotrichum* species in this complex (*C. acutatum*, *C. fioriniae*, *C. godetiae*, *C. nymphaeae*, *C. salicis*, and *C. simmondsii*) that have been reported as anthracnose pathogens of strawberry, *C. miaoliense* is phylogenetically most closely related to *C. nymphaeae* and *C. simmondsii*. Morphologically, *C. miaoliense* differs from *C. nymphaeae* (CBS 515.78) in the size of conidia (16.1 ± 2.3 × 4.9 ± 0.7 µm versus 14.2 ± 1.1 × 4.1 ± 0.34 µm), the shape of conidia (one end round and one end rounded to acute in contrast to the new species, in which both ends are acute), the size of appressoria (8.7 ± 2.5 × 5.5 ± 1.0 versus 7.5 ± 1. 1 × 5 ± 0.6 µm) (Supplementary Table S2), and the shape of appressoria (*C. miaoliense* and *C. simmondsii* appressoria are elliptical to clavate, whereas the appressoria of *C. nymphaeae* are clavate or irregular in outline, entire, and have an undulate to lobate margin^12^). Compared with *C. simmondsii*, the conidia of *C. miaoliense* are longer (mean length 14.2 µm versus 8.1 µm). In addition, the conidia of *C. simmondsii* are cylindrical with one end round and one end acute or both ends acute. Although the appressoria of *C. miaoliense* and *C. simmondsii* are similar in shape and L/W ratio, the appressoria of *C. simmondsii* are larger (Supplementary Table S2).

### Effect of temperature on mycelial growth

**Figure 5:**
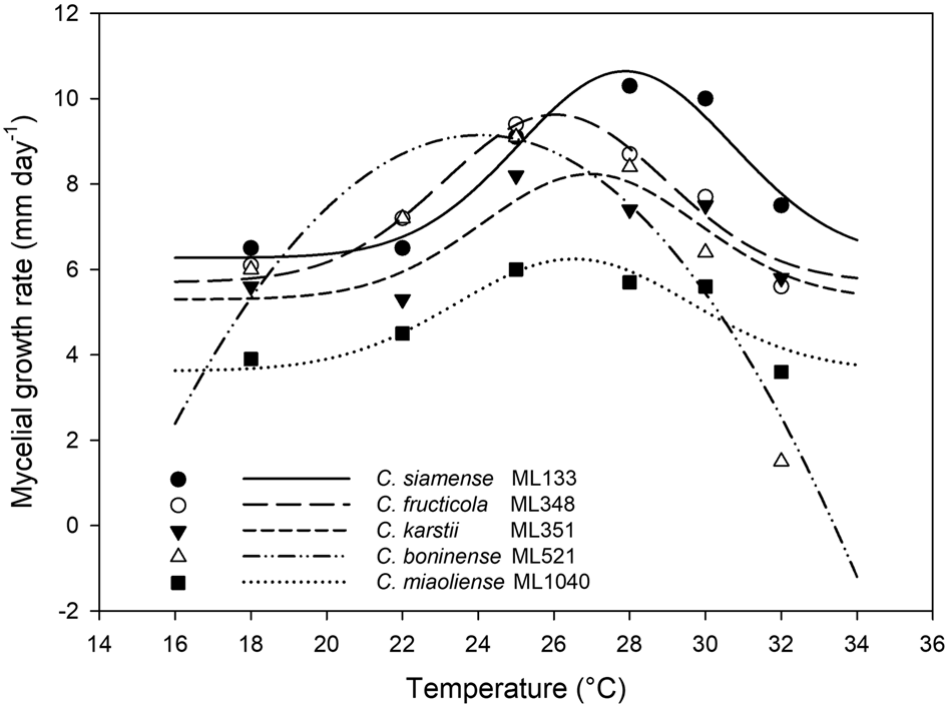
Mycelial growth rates of *Colletotrichum* spp. at different temperatures. Different symbols represent the mean growth rates of different species at the tested temperatures. Gaussian process regression was used to estimate the optimum temperature for mycelial growth.

A representative isolate selected from each of five *Colletotrichum* species was grown on PDA at 18 °C to 32 °C. The maximum growth rate of *C*. *siamense* ML133 was estimated at 27.9 °C, whereas the maximum growth rates of *C*. *fructicola* ML348, *C*. *karstii* ML351, *C*. *boninense* ML521 and *C. miaoliense* ML1040 were at 26.0 °C, 26.9 °C, 24.0 °C, and 26.5 °C, respectively (Fig. 5 and Supplementary Table S3). The growth rate of *C*. *boninense* ML521 drastically decreased at 32 °C (Fig. 5). *C. miaoliense* ML1040 exhibited the slowest growth rate at all tested temperature regimes except 32 °C (Fig. 5). The ranking of species by growth rates at higher temperatures (28 °C, 30 °C and 32 °C) is as follows: *C*. *siamense* ML133 > *C. fructicola* ML348 > *C. karstii* ML351 and *C*. *boninense* ML521 (28 °C and 30 °C) > *C. miaoliense* ML1040 > *C*. *boninense* ML521 (32 °C) (Fig. 5 and Supplementary Table S3).

The growth rates of *C*. *siamense* ML133 were also compared with those of another two representative isolates selected from *C*. *siamense*, *C*. *fructicola*, *C*. *karstii*, and *C. miaoliense* (Supplementary Table S4). The ranking of isolates by mycelial growth rates at both 25 °C and 30 °C on PDA was as follows: *C*. *siamense* ML133 and ML540 > *C*. *siamense* ML612 and *C. fructicola* ML368 > *C. fructicola* ML356 and *C. karstii* ML1792 > *C. karstii* ML442 > *C. miaoliense* ML1042 and ML1794.

### Pathogenicity assay

Pathogenicity was tested using Koch's postulates for *C. siamense* ML133, *C. fructicola* ML348, *C*. *karstii* ML351, *C*. *boninense* ML521, and *C. miaoliense* ML1040. These isolates all caused leaf and/or crown necrosis in strawberry seedlings (Fig. S6). *C. siamense* ML133 caused the most severe symptoms with 100% disease incidence. The disease incidences for *C. fructicola* ML348, *C*. *karstii* ML351, *C*. *boninense* ML521, and *C. miaoliense* ML1040 were only 30%, 30%, 30% and 50%, respectively. Notably, after spray inoculation of the seedlings with *C*. *karstii* ML351, leaf spots scarcely occurred, and no leaf lesions were observed for *C*. *boninense* ML521. Even though there were few visible symptoms, *C*. *boninense* ML521 could be re-isolated from surface-sterilized inoculated leaves.

**Figure 6:**
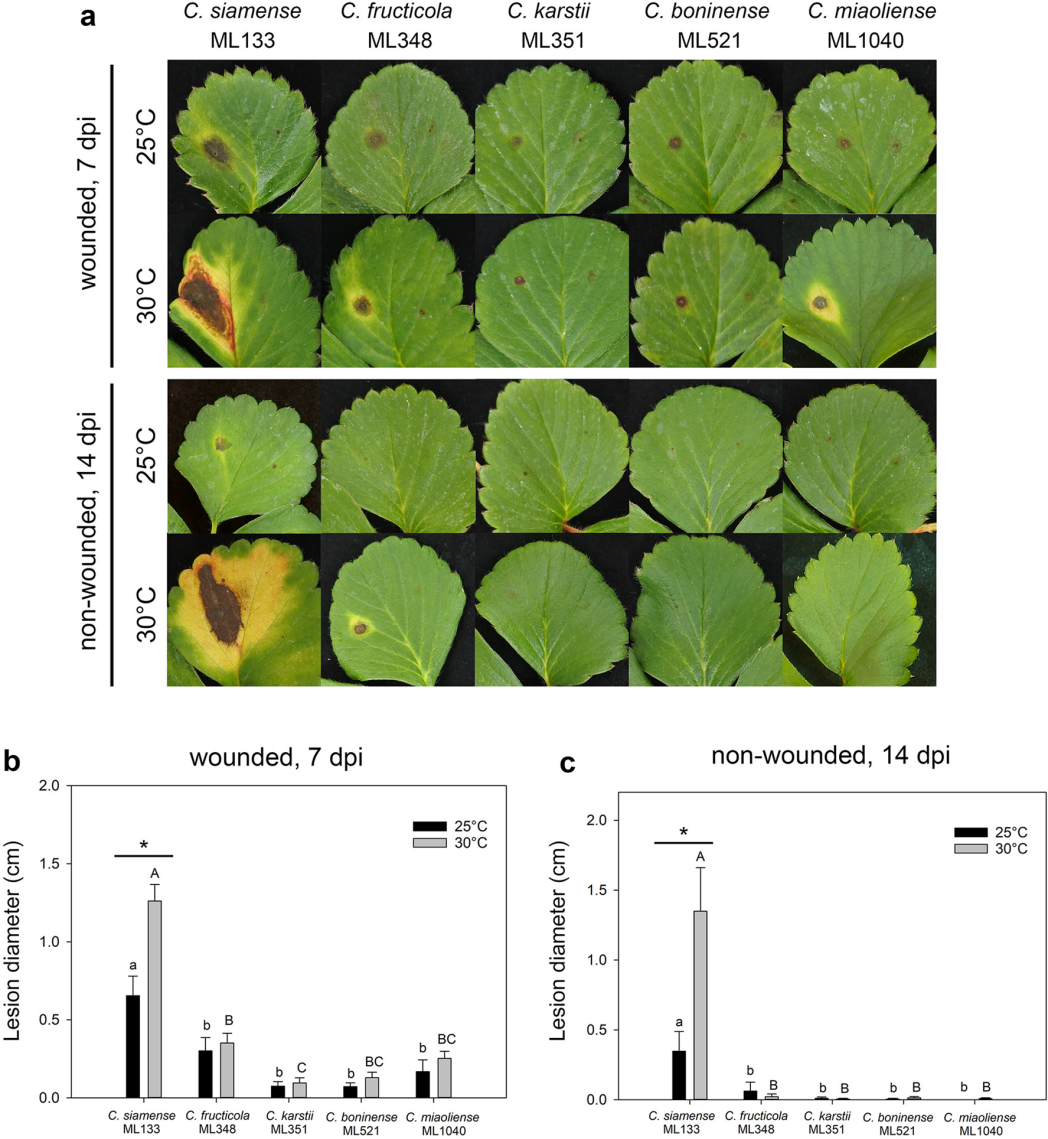
Inoculation of *Colletotrichum* spp. on detached wounded and non-wounded strawberry leaves at different temperatures. (**a**) Lesions at 7 days post inoculation (dpi) on wounded leaves or at 14 dpi on non-wounded leaves incubated at 25 °C or 30 °C. The left side of each leaflet was inoculated with 10 μl spore suspension (10^6^ spores/ml) and the right side with water (control). (**b**–**c**) Lesion sizes resulting from inoculation of wounded and non-wounded leaves. The results from the same temperature were analyzed together. Data (mean ± standard error) with different letters are significantly different based on Tukey's range test at *P* < 0.05 (n = 12). For each species, the difference between 25 °C and 30 °C was analyzed by Student’s *t* test (*denotes *P* < 0.05).

Virulence of the five selected isolates was subsequently assayed using wounded and non-wounded detached leaves at 25 °C and 30 °C (Fig. 6). For all five isolates, inoculation of wounded leaves resulted in typical anthracnose lesions, which were first observed at 2–4 days post inoculation (dpi). *C*. *siamense* ML133 caused the largest brown necrotic lesions, sometimes with chlorotic or reddish margins (Fig. 6a). The necrotic lesions caused by *C*. *fructicola* ML348, *C*. *karstii* ML351, *C*. *boninense* ML521, and *C. miaoliense* ML1040 were significantly smaller (Fig. 6b; *C*. *fructicola* ML348 was slightly more virulent than *C*. *karstii* ML351, *C*. *boninense* ML521, and *C. miaoliense* ML1040). At 7 dpi, *C*. *siamense* ML133 caused significantly larger lesions at 30 °C (1.26 cm in diameter) than 25 °C (0.65 cm in diameter), whereas the sizes of lesions caused by other *Colletotrichum* species were similar (0.07–0.35 cm in diameter) at different temperatures (Fig. 6b).

In regard to inoculations of unwounded leaves, necrotic lesions caused by *C*. *siamense* ML133 and *C*. *fructicola* ML348 first appeared at 4–7 dpi, but no lesions occurred in the plants inoculated with the other three *Colletotrichum* spp. isolates (Fig. 6a). Inoculation of unwounded leaves with *C*. *siamense* ML133 resulted in larger lesions than inoculation with the other *Colletotrichum* spp., and the lesion sizes at 14 dpi were significantly larger at 30 °C (1.35 cm in diameter) than at 25 °C (0.35 cm in diameter) (Fig. 6c).

Inoculations of wounded leaves were conducted at 30 °C for *C. siamense* ML133, *C. boninense* ML521, and two additional representative isolates of *C. siamense*, *C. fructicola*, *C. karstii*, and *C. miaoliense* (Fig. S7). The results showed that *C. siamense* (ML133, ML540, ML612) and *C. fructicola* ML356 caused significantly larger lesions (1.21–1.74 cm in diameter at 7 dpi; 3.06–3.47 cm in diameter at 14 dpi) than *C. fructicola* ML368, *C. karstii* (ML442, ML1792), and *C. miaoliense* (ML1042, ML1794) (0.42–0.76 cm in diameter at 7 dpi; 1.06–2.15 cm in diameter at 14 dpi).

## Discussion

Over the past decade, our knowledge of fungi and their relationships with plant hosts has seen an exponential growth due to the progress in bioinformatics and molecular phylogenetics. Cryptic taxa identification is progressing rapidly and groups of fungi, including important plant pathogens, are now mainly classified using molecular data-based phylogenetic inference. For instance, the *C. acutatum*, *C. boninense*, and *C. gloeosporioides* species complexes now each contain over 20 species^12,13,21^. Several *Colletotrichum* spp. with the capacity to cause strawberry anthracnose in temperate regions have been reported^12–15,17,22–30^; however, knowledge of the composition of the pathogen populations in tropical and subtropical regions is limited. Through morphological characterization, phylogenetic analyses involving five to seven loci (ITS, *GAPDH*, *CHS-1*, *ACT*, *TUB2*, *CAL*, and ApMAT), and inoculation tests on strawberry seedlings and detached leaves, the present study revealed that five *Colletotrichum* species cause strawberry anthracnose in Taiwan. In addition to the known strawberry anthracnose pathogens *C. boninense*^15^, *C. fructicola*^25,31^, and *C. siamense*^13,16,18^, one new species, *C. miaoliense*, and one species not previously known to infect strawberry, *C*. *karstii*, were identified. *C*. *karstii* was previously isolated from a wide range of plants such as anthurium, apple, citrus, and chili pepper^21,32,33^, but not from strawberry. In this study, *C. siamense*, *C. fructicola*, and *C miaoliense* were isolated from different tissues, and all five *Colletotrichum* species were proved to be pathogenic to strawberry leaves and crowns (Fig. S6). The lack of tissue specificity is in agreement with previous observations of *C. acutatum* in strawberry^2,34^.

The predominance of *C*. *siamense* (75%) and *C*. *fructicola* (11%) in the strawberry anthracnose pathogen population in Taiwan can be attributed to their higher levels of pathogenicity and aggressiveness. While all five *Colletotrichum* spp. were pathogenic to wounded leaves, only *C*. *siamense* and *C*. *fructicola* were able to cause lesions on non-wounded leaves. In addition, *C*. *siamense* (ML133, ML540, ML612) and *C*. *fructicola* (ML356) caused significantly larger lesions at 25 °C and 30 °C (Fig. 6 and Fig. S7). A similar phenomenon was observed for *Colletotrichum* spp. causing strawberry anthracnose in Zhejiang, China (latitude ~ 30°N)^18^: *C*. *fructicola* (53% of the isolates) and *C*. *siamense* (23%) dominated the population, and *C*. *fructicola* exhibited the highest level of pathogenicity (only 25 °C was tested)^18^. Although *C. boninense*, *C. karstii*, and *C. miaoliense* were much less virulent, wounds caused by natural agencies (wind, rain, insects and animals) as well as human activities (e.g., trimming old leaves, which is a common agricultural practice employed by most strawberry farmers in Taiwan) would provide potential infection sites allowing these pathogens to bypass the first line of defense (e.g., the cuticle)^35–37^ in strawberry.

Among the five *Colletotrichum* species identified in this study, *C*. *siamense* exhibited greater mycelial growth rates on PDA, especially at higher temperatures. The fitness advantage of *C*. *siamense* in warm temperature weather may explain its current geographical distribution. In the literature, *C*. *siamense* was most reported in tropical and subtropical regions^13,16,38^, whereas *C. boninense*, *C. fructicola*, and *C*. *karstii* were reported in regions across a wide range of latitudes^13,20,21,33,39–41^. Temperature is among the key environmental factors affecting a pathogen’s survival^42^. A recent study based on published observations of 612 crop pests and pathogens from 1960 onwards revealed significant positive latitudinal shifts of many important pests and pathogens under climate change^43^. More research on the genetic and biological characteristics of different *Colletotrichum* species from diverse geographical areas will be needed to understand the emergence and spread of anthracnose diseases. With rising global temperatures, it will be particularly important to monitor the expansion of the heat-adapted *C. siamense* toward high latitudes.

*C. boninense*, *C. fructicola*, *C. siamense*, and *C*. *karstii* have been isolated from diverse plants other than strawberry in different countries/regions^13,21^. In Taiwan, *C. fructicola* has been reported as an anthracnose pathogen in mango, wax apple, and chili^44,45^, *C. siamense* in lychee, star fruit, and mango^46,47^, *C. karstii* in passion fruit^48^, and *C. boninense* in pitaya^49^. Previous studies have demonstrated that *Colletotrichum* spp. from strawberry are pathogenic to other crops and even weeds. For example, *C. acutatum* could not only infect pepper, eggplant, tomato, and bean but also latently colonize weeds such as *Vicia* spp. and *Conyza* spp.^50^. In one study, *C. fructicola* was frequently isolated from leaves of *Amaranthus blitum*, and artificial inoculation of *C. fructicola* caused brown leaf spots on *A. blitum*^31^. To determine whether weed control is necessary to minimize the primary infection in the field, it is worth investigating whether the five *Colletotrichum* spp. we identified could colonize the weeds commonly present in and nearby strawberry fields in Taiwan. More sampling and artificial inoculation assays will be required to understand the host range of the new species *C. miaoliense*.

Anthracnose is a key limiting factor for strawberry production in Taiwan and many other areas. Outbreaks of anthracnose in strawberry nurseries and fields have caused yield losses of up to 50–80%^2,18,51,52^. This study demonstrated the diversity of pathogenic *Colletotrichum* species associated with strawberry in Taiwan. The findings offer precise information about pathogen identity, which is valuable for screening of resistant varieties and development of effective disease management strategies. Regardless of whether it was inoculated on wounded or non-wounded leaves, the predominant pathogen *C*. *siamense* caused larger lesions at 30 °C than 25 °C, which is meaningful in subtropical Taiwan and areas with a similar phenology. Because no significant difference was observed between the mycelial growth rates of *C*. *siamense* at 25 °C, 28 °C, and 30 °C, higher disease severity at 30 °C could be due to reduced resistance of strawberry against anthracnose at higher temperatures^53,54^. In Taiwan, the susceptible cultivar ‘Taoyuan No. 1’ has been widely cultivated for over 30 years. Development of temperature-independent resistant cultivars will be particularly important for strawberry breeding programs in Taiwan and other tropical and subtropical regions. Future work will focus on monitoring pathogen population changes, investigating the fungicide sensitivity levels of different *Colletotrichum* species, and developing molecular detection methods to aid the production of strawberry seedlings without latent infection of major *Colletotrichum* species.

## Methods

### Sample collection and pathogen isolation

From 2010 to 2018, different strawberry tissues (including the leaf, stolon, fruit, root and crown) showing anthracnose disease symptoms were collected from 24 farms and nurseries located in Miaoli, Hsinchu, Nantou, and Chiayi Counties in Taiwan. From 2009 to 2018, the strawberry-cultivated areas in Miaoli, Hsinchu, Nantou, and Chiayi accounted for approximately 89.6%, 2.8%, 2.6%, and 0.2% of the total strawberry-cultivated area in Taiwan, respectively. Pure cultures of all fungal isolates were obtained by the single hyphal tip isolation method. Approximately 2 × 2 mm fragments bordering healthy and necrotic zones in diseased tissues were surface-sterilized with 0.5–1% sodium hypochlorite, rinsed with sterile deionized water three times, then placed onto 1.5% water agar. After 2–3 days of incubation at 25 °C, single hyphal tips were transferred to potato dextrose agar (PDA, BD Difco) and cultured for further use. A total of 52 *Colletotrichum* spp. isolates were used in this study (Table 1): 26 (50%) isolated from crowns, 11 (21.2%) from leaves, 5 (9.6%) from fruits, 5 (9.6%) from roots, and 5 (9.6%) from stolons (Table 1). Type specimens in this study were deposited in the herbarium of the Department of Plant Pathology and Microbiology, National Taiwan University (NTUH). Ex-type living cultures were deposited in the Culture Collection of the Department of Plant Pathology and Microbiology, National Taiwan University (NTUCC), Bioresource and Collection Research Center (BCRC), and Miaoli District Agricultural Research and Extension Station. Nomenclature and taxonomic information were deposited in MycoBank^55^ (www.mycobank.org). *Colletotrichum* spp. were preserved as mycelium discs in ddH_2_O at 4 °C for short-term storage and in 10% glycerol with 5% lactose at − 80 °C for long term storage. Before conducting experiments, each isolate was revived by culturing on PDA for 5–7 days at 25 °C under a 12-h/12-h photoperiod.

### DNA extraction, PCR amplification, and sequencing

For each *Colletotrichum* spp. isolate, the mycelium was taken from a 7-day-old culture grown on PDA medium. The mycelium was frozen in liquid nitrogen and ground into a fine powder using a sterile mortar and pestle. Genomic DNA was extracted using the Plant Genomic DNA Extraction Miniprep System Kit (VIOGENE) according to the manufacturer’s instructions. Seven genetic fragments, namely ITS, *GAPDH*, *CHS-1*, *ACT*, *TUB2*, *CAL*, and ApMAT, were amplified with the primers listed in Supplementary Table S5 using the Biometra Thermal Cycler (Biometra TRIO). Each PCR reaction contained 1 μl of genomic DNA (20–50 ng), 5 μl of 10X reaction buffer [with Tris–HCl (pH 9.0), PCR enhancers, (NH_4_)_2_SO_4_, 20 mM MgCl_2_], 2 μl of dNTPs (2.5 mM each), 1 µl of 10 μM forward primer, 1 µl of 10 μM reverse primer, 0.5 μl (2.5 U) of Prime Taq DNA Polymerase (GenetBio Inc.), and 39.5 μl of ddH_2_O. The thermal cycling parameters were 1 cycle of 95 °C for 4 min and 30–35 cycles of 95 °C for 30 s, 52–62 °C for 30 s, and 72 °C for 60 s followed by a final extension step of 72 °C for 7 min. The optimal annealing temperatures for different genetic regions were: ITS: 58 °C, *GAPDH*: 52 °C, *CHS-1*: 58 °C, *ACT*: 58 °C, *TUB2*: 58 °C, *CAL*: 59 °C, and ApMAT: 62 °C. Amplicons were bidirectionally sequenced using the dideoxy termination method on the ABI 3730 DNA Analyzer (Tri-I Biotech, Taiwan). Raw sequence chromatograms were manually examined, and the sequences of each fragment were assembled in ApE v2.0.55 (A Plasmid Editor, M. Wayne Davis at the University of Utah, Salt Lake City, UT).

### Multilocus phylogenetic analysis and species recognition

Newly generated sequences from each isolate were blasted against the GenBank nr database, and searches were restricted to type materials for initial determination of the closest matching species and species complex. Related gene sequences (ITS, *GAPDH*, *CHS-1*, *ACT*, *TUB2*, *CAL*, and ApMAT) of *Colletotrichum* spp. from recent publications were downloaded from GenBank^12,13,21^. For each gene, sequences from the isolates belonging to the same species complex were aligned using the MAFFT v7 online server (https://mafft.cbrc.jp/alignment/server/)^56^. The aligned sequences were manually edited using MEGA v10^57^ to improve the alignment. The post-alignment sequences of multiple genes/loci were concatenated in a text editor.

BI, ML, and MP approaches for each individual locus and the concatenated sequences were used to identify closely related taxa. Best-fit models of nucleotide substitution were selected using the Akaike information criterion implemented in MrModeltest v.2.4^58^ and run in PAUP v.4.0^59^ (Supplementary Table S6). BI analyses were performed using MrBayes v.3.2.6^60^. Two independent analyses of four Markov Chain Monte Carlo (MCMC) chains (3 heated, 1 cold) were run from a random tree for 2 × 10^6^ (for the *C*. *acutatum* species complex), 4 × 10^6^ (for the *C*. *boninense* species complex), and 6 × 10^6^ (for the *C*. *gloeosporioides* species complex) generations or until the average standard deviation of split frequencies was below 0.01. The analysis was sampled every 1,000 generations, and the first 25% of the generations were discarded as burn-in. The effective sample size and convergence were monitored with Tracer v1.7.1^61^. MP analyses were performed in PAUP v.4.0^59^ using the heuristic search option with Tree Bisection Reconnection branch swapping and 100 random sequence addition. Maxtrees were set to 5,000 and bootstrap analysis was performed with 1,000 replicates. ML analyses were performed in RAxML v8.2.10^62^ using the GTR-gamma substitution model with 1,000 bootstrap replicates. Phylogenetic trees were visualized in FigTree v1.4.3. The concatenated alignments and phylogenetic trees were deposited in TreeBASE (www.treebase.org) with the study ID 26665.

We applied the Genealogical Concordance Phylogenetic Species Recognition (GCPSR) method^63,64^ for species delimitation of *Colletotrichum* taxa. A novel species was considered novel the clade was strongly supported as monophyletic by BI (posterior probability ≥ 0.95), ML (bootstrap ≥ 70%), and MP (bootstrap ≥ 70%) analyses in the multilocus phylogenetic tree and in the majority of individual gene trees.

### Morphological characterization

Morphological characterization of one selected isolate from each *Colletotrichum* species was conducted following the procedures of Weir et al. (2012) and Damm et al. (2012)^12,13,21^. Cultures were grown on PDA and 1/4-strength PDA (1/4 PDA)^65^ for 2–3 weeks at 25 °C and 30 °C under a 12-h/12-h photoperiod. (Our preliminary tests showed that compared with PDA, the low nutrient medium 1/4 PDA could stimulate sporulation without affecting the size and shape of conidia.) The experiment was performed in two independent trials, each consisting of two to three plates per isolate. Conidiomata were investigated using a dissecting microscope (Leica M125). Conidia, conidiophores, setae, asci, ascomata and appressoria were examined using a light microscope (Leica DM2500). To induce the formation of appressoria, 20–30 µl of conidial suspension (prepared using sterile dH_2_O) was dropped onto a microscope slide, covered with a cover slip, then incubated in a moist chamber at 25 °C for 2–3 days^39^. The lengths and widths of 55 conidiogenous cells, 100 conidia and 30 appressoria were measured using ImageJ software^66^.

### Effect of temperature on mycelial growth

Mycelium-agar discs (6 mm in diameter) were cut with a sterilized cork borer from the advancing edge of 5- to 7-day-old *Colletotrichum* spp. colonies then placed (with the mycelium-side down) onto the center of a 90 mm petri dish containing 25 ml PDA. Colony diameters were measured after 7 days of incubation at different temperatures under a 12-h/12-h photoperiod in a growth chamber (Firstek, GC-560H). The mycelial growth rate (mm/day) was calculated as “(the diameter of the colony—the diameter of the mycelium-agar disc) / 7”. For one selected isolate from each *Colletotrichum* species, the mycelial growth rates at 18 °C, 22 °C, 25 °C, 28 °C, 30 °C, and 32 °C were determined in two to three independent trials, each consisting of three PDA plates per isolate per temperature. The optimum temperature for the mycelial growth rate was estimated based on the Gaussian process (4 parameter) for nonlinear regression in SigmaPlot 14 (Systat Software, San Jose, CA). Growth of an additional two representative isolates of each *Colletotrichum* species, selected from distinct subclades within the species based on the multilocus phylogenetic analysis, was measured at 25 °C and 30 °C in two independent trials, each consisting of four PDA plates per isolate per temperature.

### Pathogenicity assay

The susceptible cultivar ‘Taoyuan No. 1’ was used for all inoculation tests in this study. The pathogenicity of *Colletotrichum* spp. (one isolate from each *Colletotrichum* species) was examined via Koch's postulates. Strawberry seedlings at the four- to five-leaf stage were inoculated by spraying a spore suspension on the leaves until runoff (10^6^ spores/ml), and also applying 1 ml of spore suspension (10^6^ spores/ml) on the crown region after removal of one to two old leaves. After inoculation, the seedlings were covered with plastic bags (> 90% relative humidity) for 3 days at 30 °C and then incubated in a growth chamber for 11 days at 30 °C and 70% relative humidity under a 12-h/12-h photoperiod. The fungi were re-isolated from lesions of diseased tissues (as mentioned above using the single hypha tip isolation method), then identified based on morphological characteristics and ITS sequences as described above.

The virulence levels of different *Colletotrichum* spp. isolates were determined by inoculation of wounded and non-wounded detached strawberry leaves. Fully expanded healthy leaves were collected from strawberry seedlings at the four- to five-leaf stage. For inoculation of wounded leaves, each leaflet was punctured with a sterile syringe needle on the left and right sides of the midrib, and 10 μl of a spore suspension (1 × 10^6^ spores/ml) was deposited on the left wound site, and sterile dH_2_O was deposited on the right wound site as a control. Similarly, inoculations of non-wounded leaves were performed in the same way as mentioned above. After inoculation, the leaves were kept in a moist chamber (a plastic box with dH_2_O at the bottom; the cut end of the petiole was submerged in the water) at 25 °C or 30 °C under a 12-h/12-h photoperiod. Lesion size was measured at 7 and 14 days post inoculation (dpi). Lesions smaller than 0.1 cm in diameter were considered unsuccessful infections. The same isolates for the “effect of temperature on mycelial growth” test were used for the pathogenicity assay. For one selected isolate from each *Colletotrichum* species, inoculations of wounded and non-wounded leaves were performed at 25 °C and 30 °C in three independent trials, each consisting of 12 leaflets (4 leaves from 4 seedlings) per isolate per treatment. An additional two representative isolates of each *Colletotrichum* species, selected from distinct subclades within the species based on the multilocus phylogenetic analysis, were used for wound inoculation at 30 °C. The experiment was performed in two independent trials, each consisting of 12 leaflets (4 leaves from 4 seedlings) per isolate per treatment.

### Statistical analysis

Data were analyzed by analysis of variance (ANOVA) using the software SPSS v18. Tukey’s range test or Student’s *t*-test was used to test for significant differences among or between different treatments at a significance threshold of *P* < 0.05.

## Supplementary information

Supplementary file1
