# Supplementary material for: Diversity and pathogenicity of Colletotrichum species causing strawberry anthracnose in Taiwan and description of a new species, Colletotrichum miaoliense sp. nov
Source: Sci Rep. 2020 Sep 4;10:14664. doi: 10.1038/s41598-020-70878-2 (PMC7473857; doi:10.1038/s41598-020-70878-2)
Supplement: Supplementary file 1 — Supplementary file1 [file 41598_2020_70878_MOESM1_ESM.pdf]

Diversity and pathogenicity of *Colletotrichum* species causing strawberry anthracnose in Taiwan and description of a new species, *Colletotrichum miaoliense* sp. nov.

Pei-Che Chung[1,2], Hung-Yi Wu[2], Yen-Wen Wang[2], Hiran-A. Ariyawansa[2], Hsien-Pin Hu[2], Ting-Hsuan Hung[2†], Shean-Shong Tzean[2†], Chia-Lin Chung[2†]

[1]Miaoli District Agricultural Research and Extension Station, Council of Agriculture, Executive Yuan, Miaoli County 36346, Taiwan

[2]Department of Plant Pathology and Microbiology, National Taiwan University, Taipei City 10617, Taiwan

†Corresponding Authors:

T.-H. Hung: +886-2-33664600; thhung@ntu.edu.tw

S.-S. Tzean: +886-2-33664595; sst@ntu.edu.tw

C.-L. Chung: +886-2-33664597; clchung@ntu.edu.tw

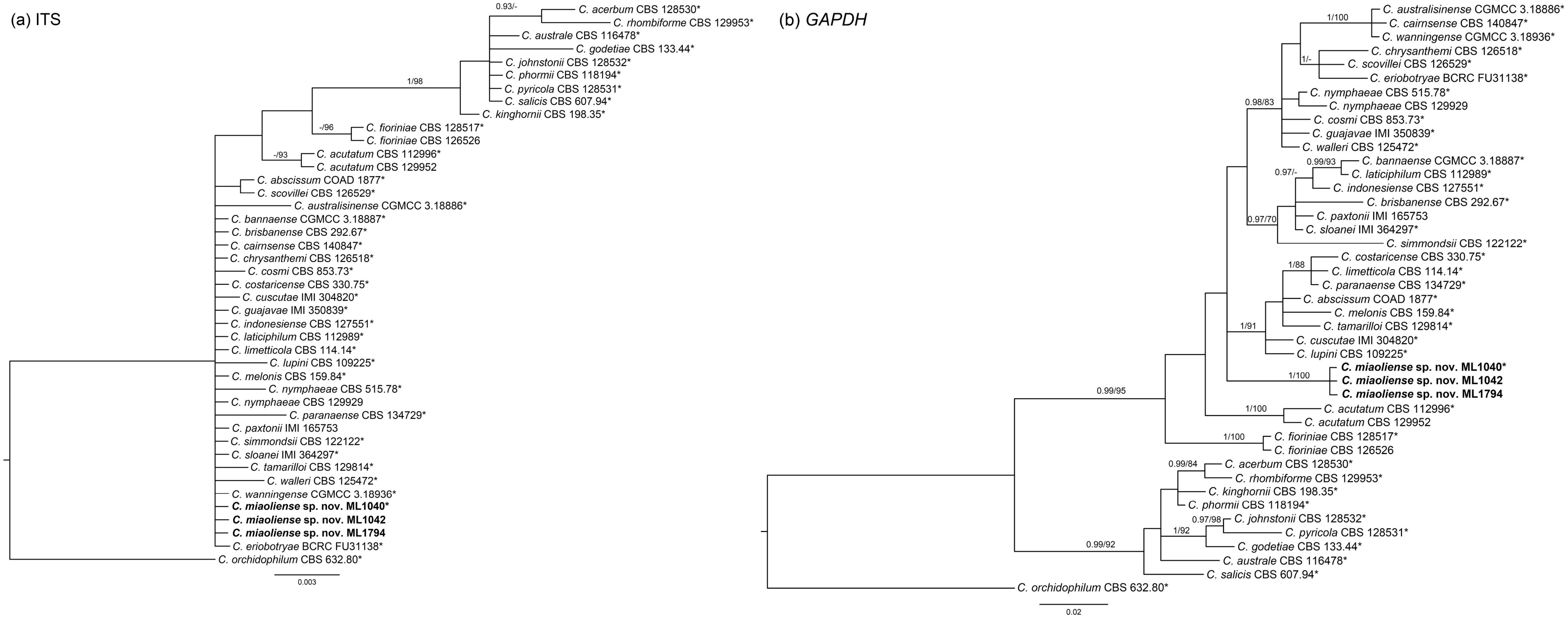

(a) ITS
0.93/-
C. acerbum CBS 128530*
C. rhombiforme CBS 129953*
C. australe CBS 116478*
C. godetiae CBS 133.44*
C. johnstonii CBS 128532*
1/98
C. phormii CBS 118194*
C. pyricola CBS 128531*
C. salicis CBS 607.94*
C. kinghornii CBS 198.35*
-/96
C. fioriniae CBS 128517*
C. fioriniae CBS 126526
-/93
C. acutatum CBS 112996*
C. acutatum CBS 129952
C. abscissum COAD 1877*
C. scovillei CBS 126529*
C. australisinense CGMCC 3.18886*
C. bannaense CGMCC 3.18887*
C. brisbanense CBS 292.67*
C. cairnsense CBS 140847*
C. chrysanthemi CBS 126518*
C. cosmi CBS 853.73*
C. costaricense CBS 330.75*
C. cuscutae IMI 304820*
C. guajavae IMI 350839*
C. indonesiense CBS 127551*
C. laticiphilum CBS 112989*
C. limetticola CBS 114.14*
C. lupini CBS 109225*
C. melonis CBS 159.84*
C. nymphaeae CBS 515.78*
C. nymphaeae CBS 129929
C. paranaense CBS 134729*
C. paxtonii IMI 165753
C. simmondsii CBS 122122*
C. sloanei IMI 364297*
C. tamarilloi CBS 129814*
C. walleri CBS 125472*
C. wanningense CGMCC 3.18936*
C. miaoliense sp. nov. ML1040*
C. miaoliense sp. nov. ML1042
C. miaoliense sp. nov. ML1794
C. eriobotryae BCRC FU31138*
C. orchidophilum CBS 632.80*
0.003
(b) GAPDH
1/100
C. australisinense CGMCC 3.18886*
C. cairnsense CBS 140847*
C. wanningense CGMCC 3.18936*
C. chrysanthemi CBS 126518*
1/-
C. scovillei CBS 126529*
C. eriobotryae BCRC FU31138*
C. nymphaeae CBS 515.78*
0.98/83
C. nymphaeae CBS 129929
C. cosmi CBS 853.73*
C. guajavae IMI 350839*
C. walleri CBS 125472*
0.99/93
C. bannaense CGMCC 3.18887*
0.97/-
C. laticiphilum CBS 112989*
C. indonesiense CBS 127551*
C. brisbanense CBS 292.67*
C. paxtonii IMI 165753
0.97/70
C. sloanei IMI 364297*
C. simmondsii CBS 122122*
C. costaricense CBS 330.75*
1/88
C. limetticola CBS 114.14*
C. paranaense CBS 134729*
C. abscissum COAD 1877*
C. melonis CBS 159.84*
1/91
C. tamarilloi CBS 129814*
C. cuscutae IMI 304820*
C. lupini CBS 109225*
C. miaoliense sp. nov. ML1040*
1/100
C. miaoliense sp. nov. ML1042
0.99/95
C. miaoliense sp. nov. ML1794
1/100
C. acutatum CBS 112996*
C. acutatum CBS 129952
1/100
C. fioriniae CBS 128517*
C. fioriniae CBS 126526
0.99/84
C. acerbum CBS 128530*
C. rhombiforme CBS 129953*
C. kinghornii CBS 198.35*
C. phormii CBS 118194*
0.97/98
C. johnstonii CBS 128532*
1/92
C. pyricola CBS 128531*
C. godetiae CBS 133.44*
0.99/92
C. australe CBS 116478*
C. salicis CBS 607.94*
C. orchidophilum CBS 632.80*
0.02

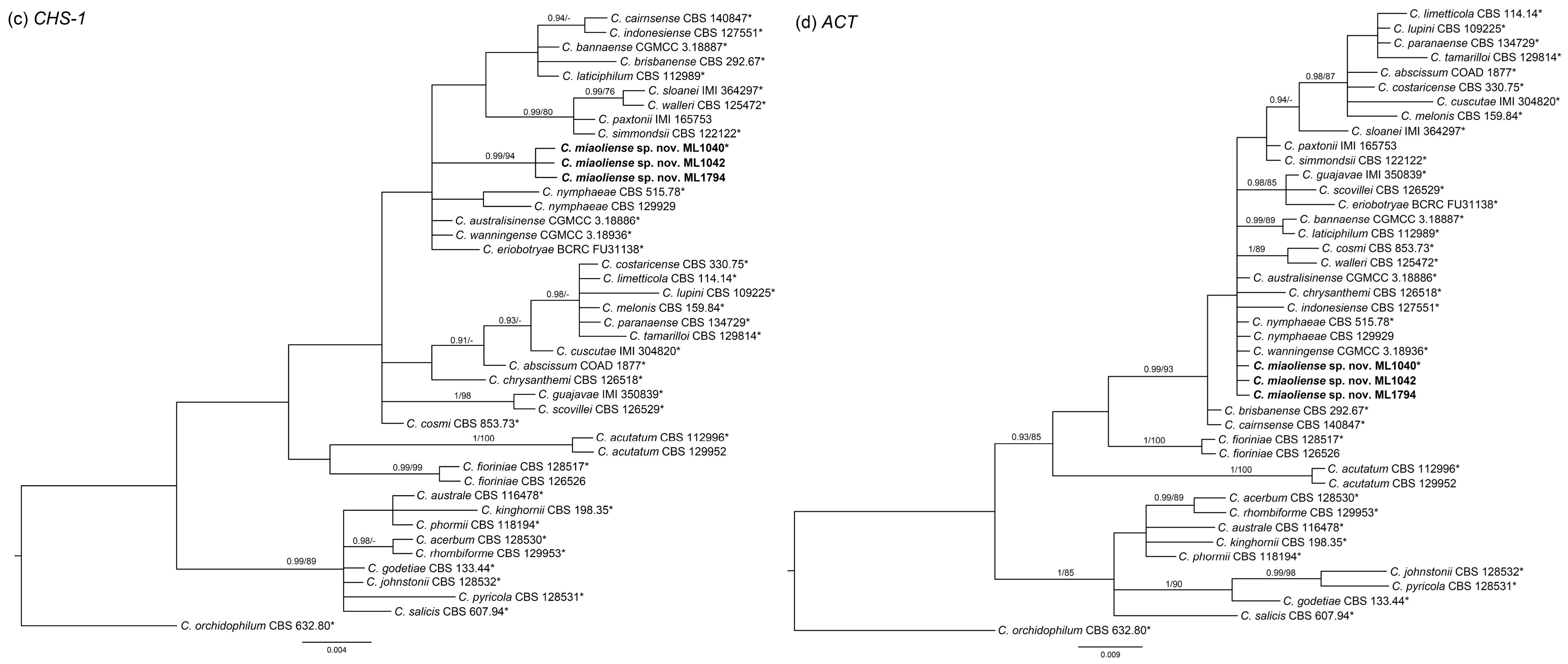
(c) CHS-1
C. cairnsense CBS 140847*
C. indonesiense CBS 127551*
C. bannaense CGMCC 3.18887*
C. brisbanense CBS 292.67*
C. laticiphilum CBS 112989*
C. sloanei IMI 364297*
C. walleri CBS 125472*
C. paxtonii IMI 165753
C. simmondsii CBS 122122*
C. miaoliense sp. nov. ML1040*
C. miaoliense sp. nov. ML1042
C. miaoliense sp. nov. ML1794
C. nymphaeae CBS 515.78*
C. nymphaeae CBS 129929
C. australisinense CGMCC 3.18886*
C. wanningense CGMCC 3.18936*
C. eriobotryae BCRC FU31138*
C. costaricense CBS 330.75*
C. limetticola CBS 114.14*
C. lupini CBS 109225*
C. melonis CBS 159.84*
C. paranaense CBS 134729*
C. tamarilloi CBS 129814*
C. cuscutae IMI 304820*
C. abscissum COAD 1877*
C. chrysanthemi CBS 126518*
C. guajavae IMI 350839*
C. scovillei CBS 126529*
C. cosmi CBS 853.73*
C. acutatum CBS 112996*
C. acutatum CBS 129952
C. fioriniae CBS 128517*
C. fioriniae CBS 126526
C. australe CBS 116478*
C. kinghornii CBS 198.35*
C. phormii CBS 118194*
C. acerbum CBS 128530*
C. rhombiforme CBS 129953*
C. godetiae CBS 133.44*
C. johnstonii CBS 128532*
C. pyricola CBS 128531*
C. salicis CBS 607.94*
C. orchidophilum CBS 632.80*
0.94/-
0.99/76
0.99/80
0.99/94
0.98/-
0.93/-
0.91/-
1/98
1/100
0.99/99
0.98/-
0.99/89
0.004
(d) ACT
C. limetticola CBS 114.14*
C. lupini CBS 109225*
C. paranaense CBS 134729*
C. tamarilloi CBS 129814*
C. abscissum COAD 1877*
C. costaricense CBS 330.75*
C. cuscutae IMI 304820*
C. melonis CBS 159.84*
C. sloanei IMI 364297*
C. paxtonii IMI 165753
C. simmondsii CBS 122122*
C. guajavae IMI 350839*
C. scovillei CBS 126529*
C. eriobotryae BCRC FU31138*
C. bannaense CGMCC 3.18887*
C. laticiphilum CBS 112989*
C. cosmi CBS 853.73*
C. walleri CBS 125472*
C. australisinense CGMCC 3.18886*
C. chrysanthemi CBS 126518*
C. indonesiense CBS 127551*
C. nymphaeae CBS 515.78*
C. nymphaeae CBS 129929
C. wanningense CGMCC 3.18936*
C. miaoliense sp. nov. ML1040*
C. miaoliense sp. nov. ML1042
C. miaoliense sp. nov. ML1794
C. brisbanense CBS 292.67*
C. cairnsense CBS 140847*
C. fioriniae CBS 128517*
C. fioriniae CBS 126526
C. acutatum CBS 112996*
C. acutatum CBS 129952
C. acerbum CBS 128530*
C. rhombiforme CBS 129953*
C. australe CBS 116478*
C. kinghornii CBS 198.35*
C. phormii CBS 118194*
C. johnstonii CBS 128532*
C. pyricola CBS 128531*
C. godetiae CBS 133.44*
C. salicis CBS 607.94*
C. orchidophilum CBS 632.80*
0.98/87
0.94/-
0.98/85
0.99/89
1/89
0.99/93
0.93/85
1/100
1/100
0.99/89
0.99/98
1/90
1/85
0.009

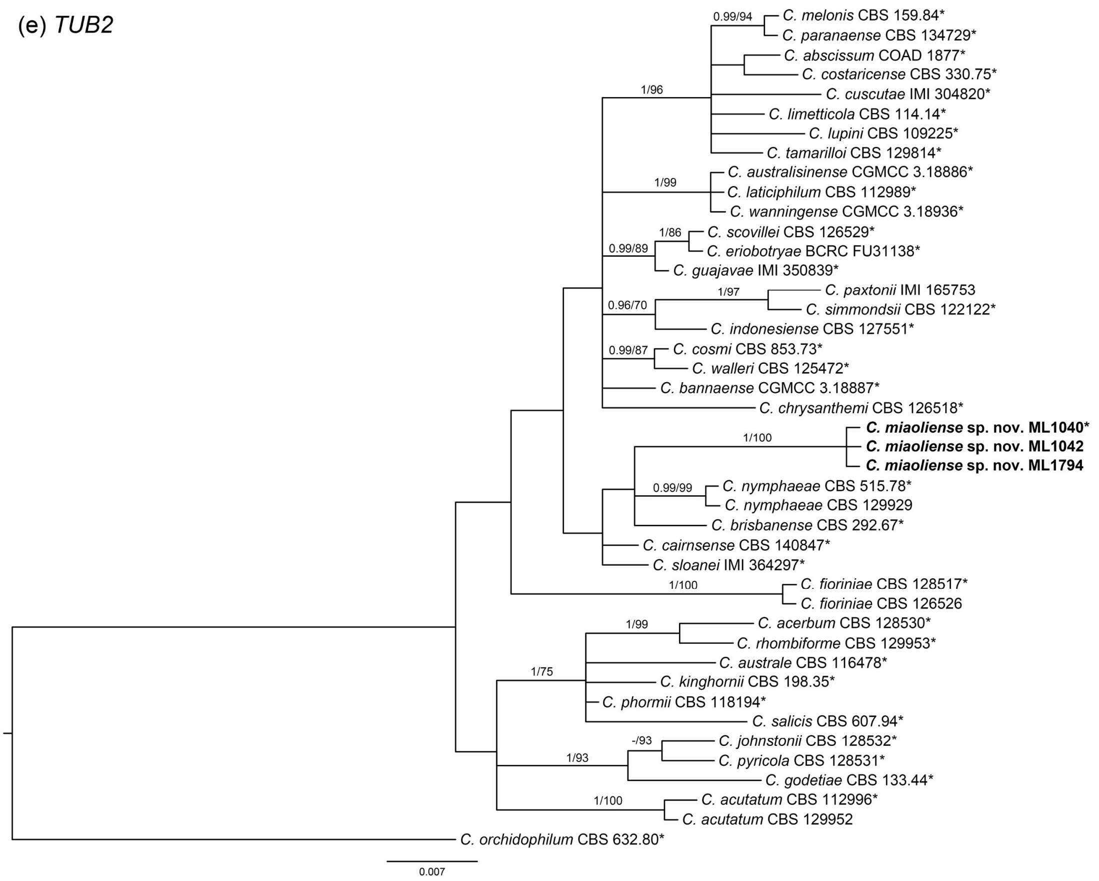

Supplementary Figure S1. Single-locus Bayesian inference phylogenetic trees of the *C. acutatum* species complex. The phylogenetic trees were built using the sequences of (a) ITS, (b) *GAPDH*, (c) *ACT*, (d) *CHS-1* and (e) *TUB2*. Bayesian inference (BI) posterior values above 0.9 and bootstrap support values from maximum likelihood (ML) above 70% are shown at each node (BI/ML). *C. orchidophilum* CBS 632.80 was used as the outgroup. * indicates the ex-type strains. Strains isolated in this study are shown in bold.

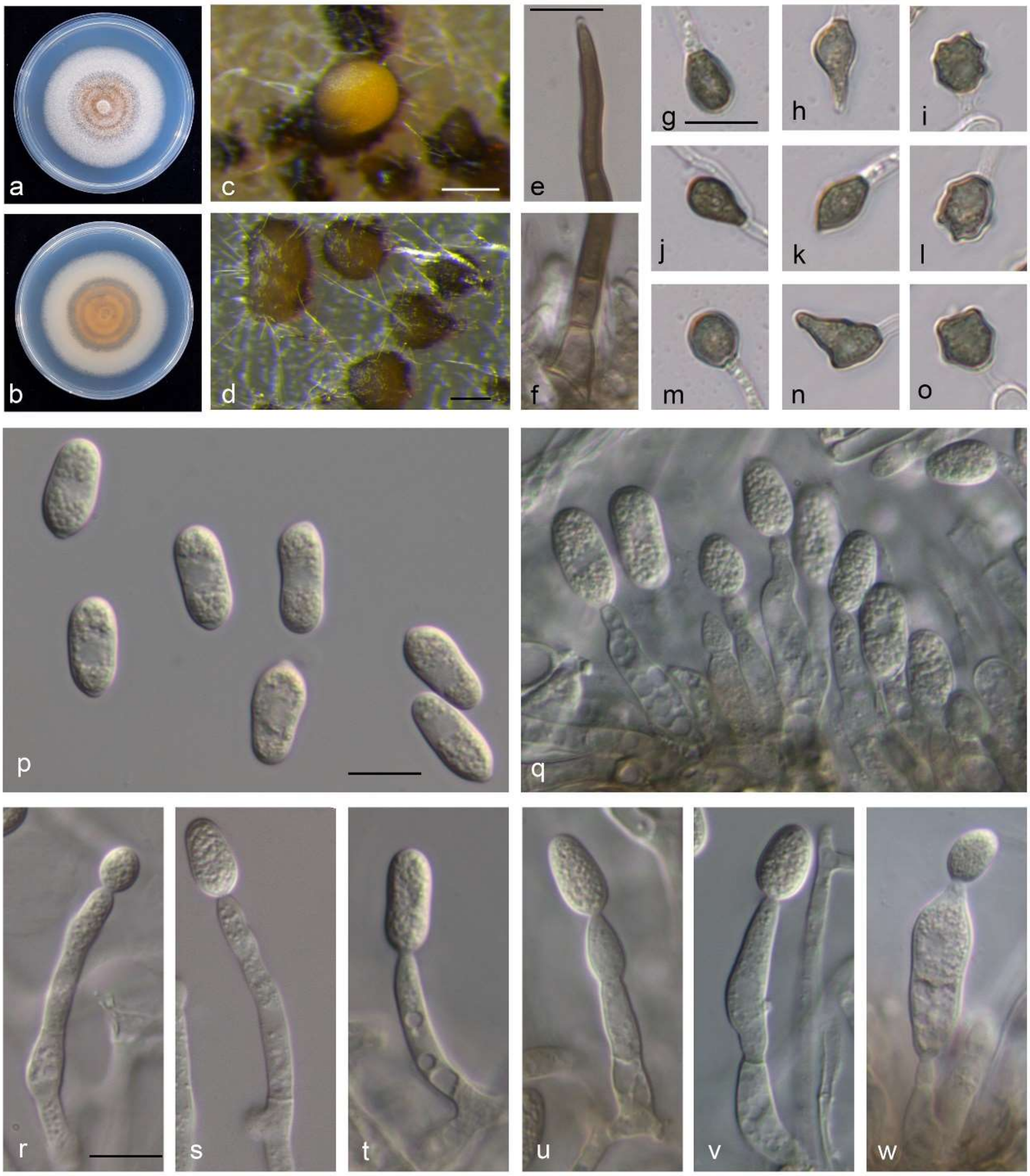

Supplementary Figure S2. ***Colletotrichum boninense*** **ML521.** (a) Upper side of colony; (b) reverse side of colony; (c-d) conidiomata; (e) tip of seta; (f) base of seta. (g-o) appressorium (induced in dH_2O on a microscope slide) [8.88 ± 1.33 × 6.12 ± 0.71 µm (n = 30)]; (p) conidia [18.23 ± 1.12 × 6.77 ± 0.54 µm (n = 100)]; (q-w) conidiophores. a-b on potato dextrose agar (PDA); c-f and p-w on 1/4-strength PDA. Scale bars: c, d = 0.2 mm; e, g, p, r = 10 µm, applies to e-w.

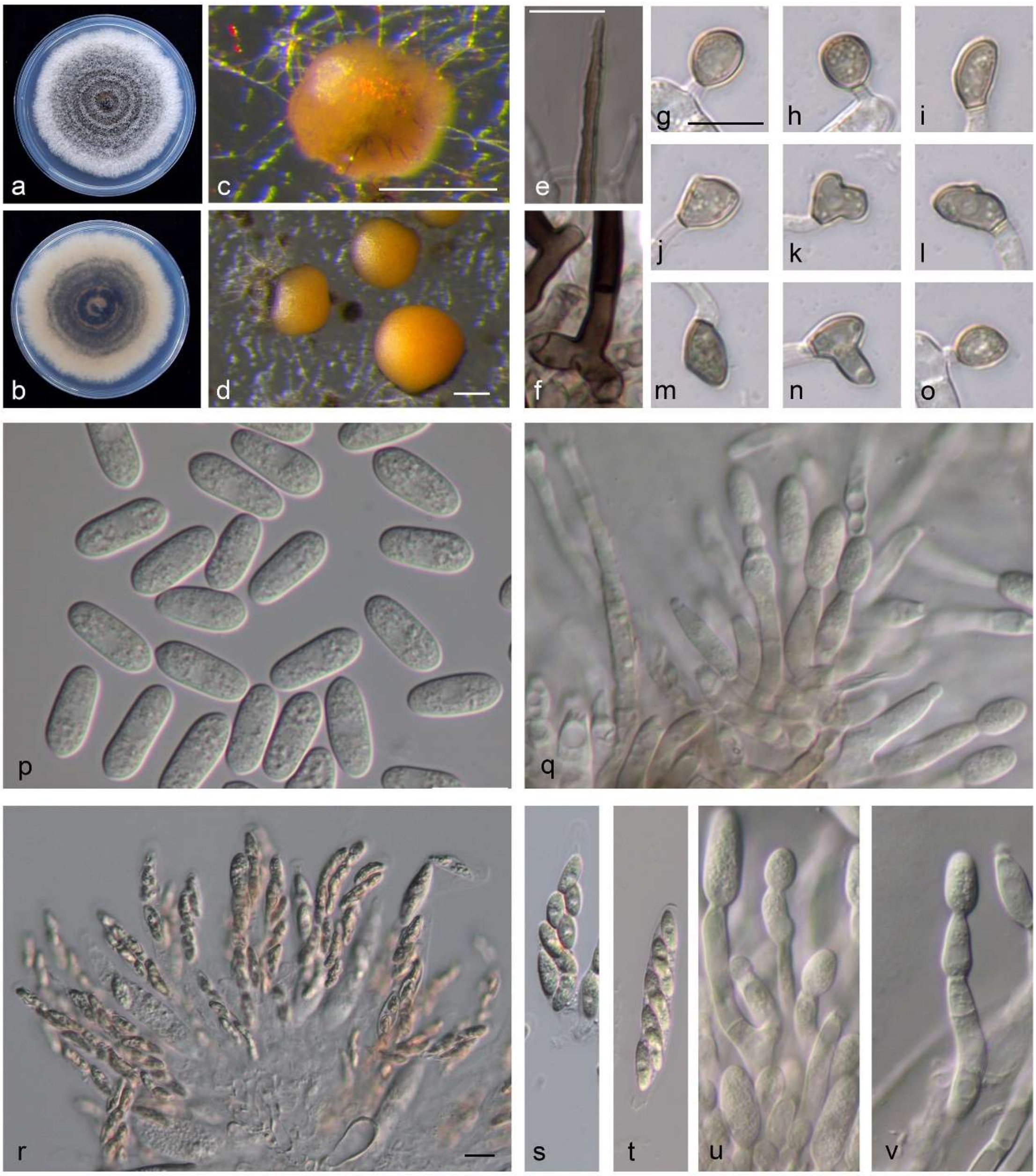

Supplementary Figure S3. ***Colletotrichum fructicola*** **ML348.** (a) Upper side of colony; (b) reverse side of colony; (c-d) conidiomata; (e) tip of seta; (f) base of seta; (g-o) appressorium (induced in dH_2O on a microscope slide) [8.09 ± 0.89 × 6.31 ± 1.03 µm (n = 30)]; (p) conidia [13.03 ± 0.69 × 5.29 ± 0.4 µm (n = 100)]; (q, u-v) conidiophores; (r-t) asci. a-b on potato dextrose agar (PDA); c-f and p-v on 1/4-strength PDA. Scale bars: c, d = 0.2 mm; e, g = 10 µm, applies to e-q, u-v; r = 10 µm, applies to r-t.

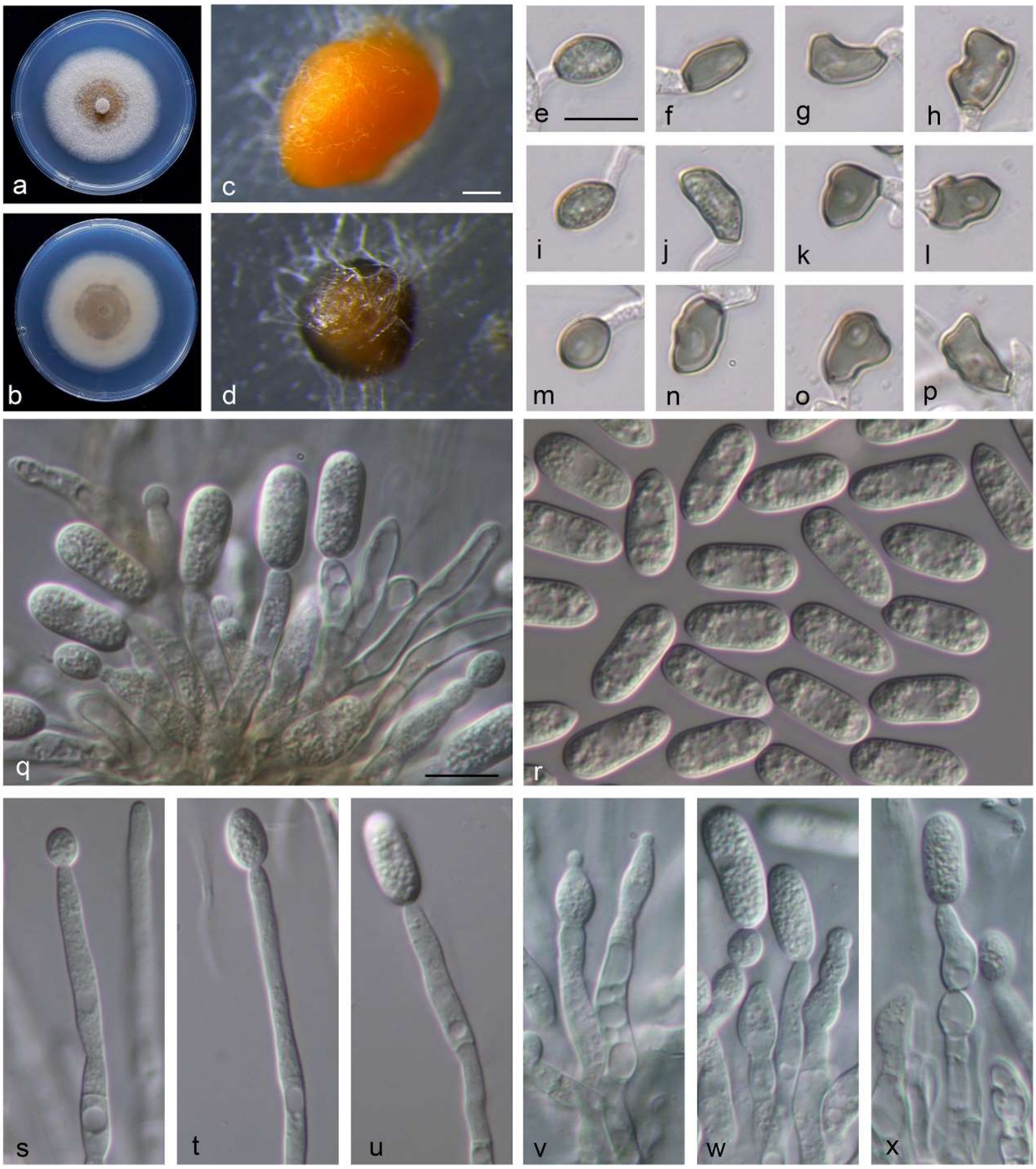

Supplementary Figure S4. ***Colletotrichum karstii*** **ML351.** (a) Upper side of colony; (b) reverse side of colony; (c-d) conidiomata; (e-p) appressorium (induced in dH_2O on a microscope slide) [8.73 ± 1.24 × 6.40 ± 0.75 µm (n = 30)]; (q, s-x) conidiophore; (r) conidia [15.53 ± 1.23 × 6.49 ± 0.36 µm (n = 100)]. a-b on potato dextrose agar (PDA); c-d and q-x on 1/4-strength PDA. Scale bars: c = 0.2 mm, applies to d; e, q = 10 µm, applies to e-x.

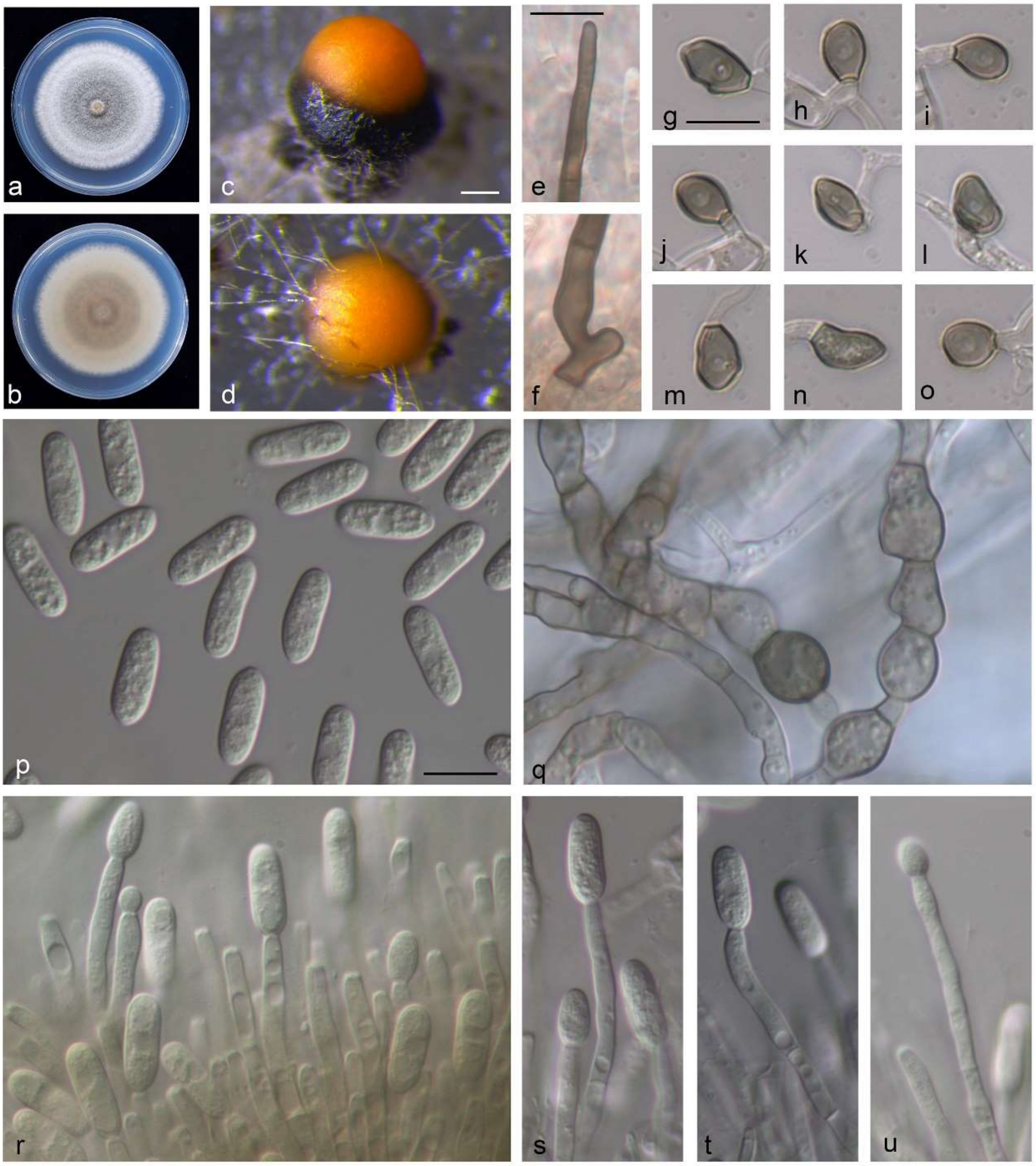

Supplementary Figure S5. ***Colletotrichum siamense*** **ML133.** (a) Upper side of colony; (b) reverse side of colony; (c-d) conidiomata; (e) tip of seta; (f) base of seta; (g-o) appressorium (induced in dH_2O on a microscope slide) [8.44 ± 0.91 × 5.73 ± 0.47 (n = 30)]; (p) conidia [14.47 ± 0.88 × 4.88 ± 0.27 µm (n = 100)]; (q) thick-walled mycelium; (r-u) conidiophore. a-b on potato dextrose agar (PDA); c-f and p-u on 1/4-strength PDA. Scale bars: c = 0.2 mm, applies to d; e, g, p = 10 µm, applies to e-u.

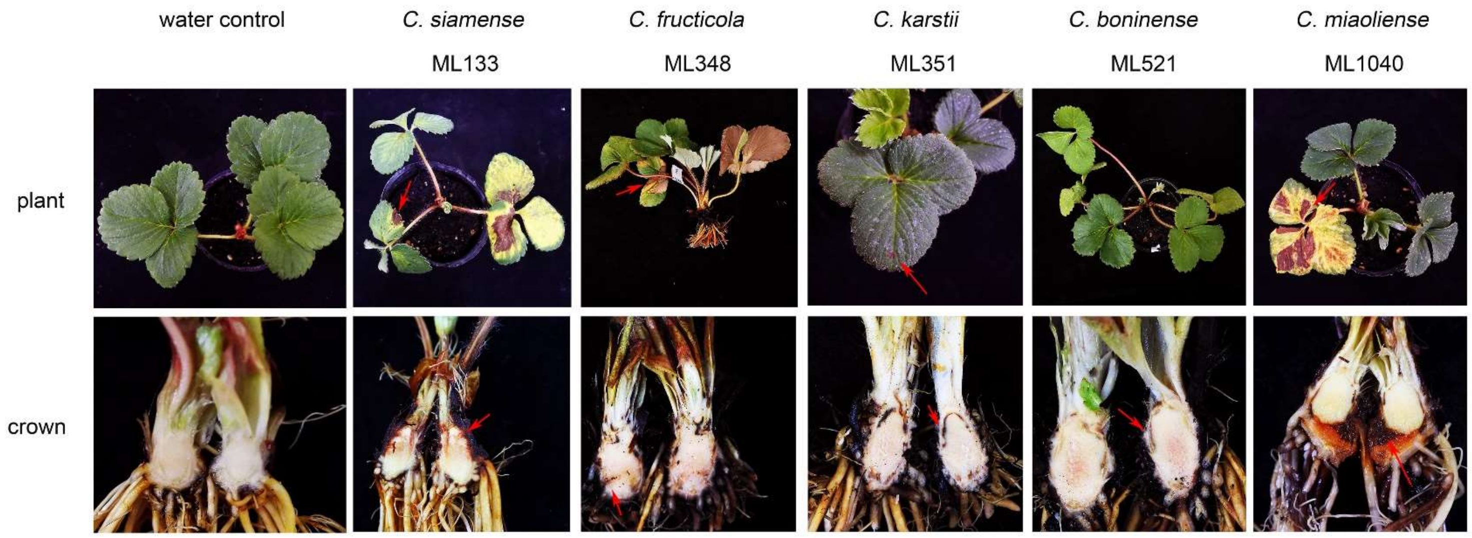

Supplementary Figure S6. Leaf necrosis and crown rot symptoms caused by *Colletotrichum* spp. Strawberry seedlings were spray-inoculated with spore suspension on the leaves and treated with spore suspension on the crown after removal of old leaves. Water was used as a control. The photos were taken at 14 days post inoculation.

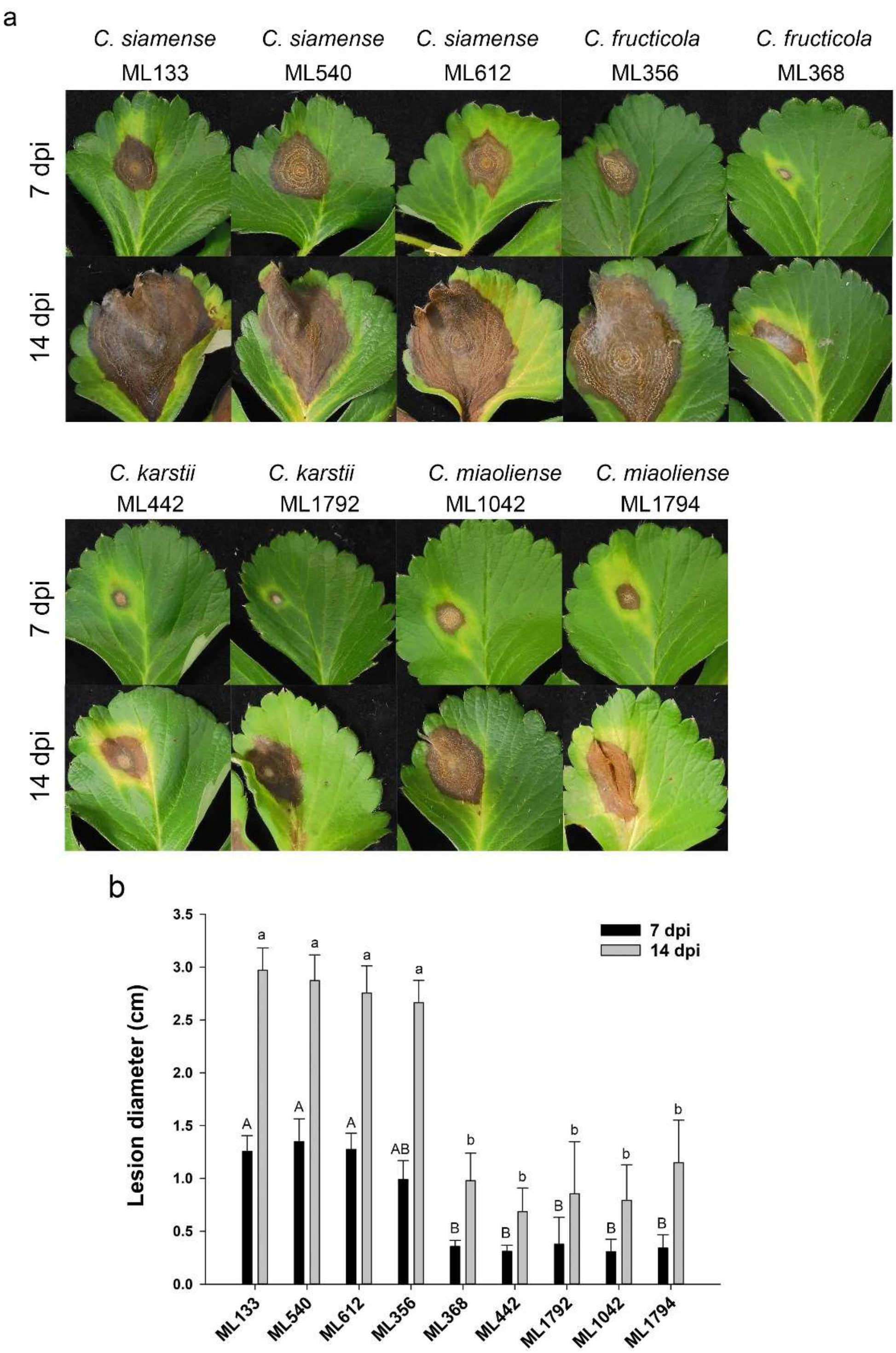

Supplementary Figure S7. Inoculation of representative isolates of different *Colletotrichum* spp. on detached strawberry leaves at 30°C. (a) Lesions at 7 and 14 days post inoculation (dpi) on wounded leaves. The left side of each leaflet was inoculated with 10 μl spore suspension (10^6 spores/ml) and the right side with water (control). (b) Lesion sizes. The results from the same day were analyzed together. Data (mean ± standard error) with different letters are significantly different based on Tukey's range test at $P < 0.05$ (n = 8).

Supplementary Table S1. List of *Colletotrichum* spp. strains used for phylogenetic analysis

Species	Culture[a]	Host/Tissue	Country/Region	GenBank accession number[b]							Ref.
				ITS	*GAPDH*	*CHS1*	*ACT*	*TUB2*	*CAL*	ApMAT	
C. acutatum species complex											
C. abscissum	COAD 1877*	*Citrus sinensis* cv. Pera	Brazil	KP843126	KP843129	KP843132	KP843141	KP843135	-[c]	-	Fu et al. (2019)[1]
C. acerbum	CBS 128530*	*Malus domestica*	New Zealand	JQ948459	JQ948790	JQ949120	JQ949780	JQ950110	-	-	Fu et al. (2019)[1]
C. acutatum	CBS 112996*	*Carica papaya*	Australia	JQ005776	JQ948677	JQ005797	JQ005839	JQ005860	-	-	Fu et al. (2019)[1]
C. acutatum	CBS 129952	*Olea europaea*	Portugal	JQ948364	JQ948695	JQ949025	JQ949685	JQ950015	-	-	Guarnaccia et al. (2017)[2]
C. australe	CBS 116478*	*Trachycarpus fortunei*	South Africa	JQ948455	JQ948786	JQ949116	JQ949776	JQ950106	-	-	Fu et al. (2019)[1]
C. australisinense	CGMCC 3.18886*	*Hevea brasiliensis*	China, Guangxi	MG209623	MG241962	MG241981	MG241947	MG209645	-	-	Liu et al. (2018)[3]
C. bannaense	CGMCC 3.18887*	*Hevea brasiliensis*	China, Yunnan	MG209638	MG242006	MG241996	MG242002	MG209660	-	-	Liu et al. (2018)[3]
C. brisbanense	CBS 292.67*	*Capsicum annuum*	Australia	JQ948291	JQ948621	JQ948952	JQ949612	JQ949942	-	-	Fu et al. (2019)[1]
C. cairnsense	CBS 140847*	*Capsicum annuum*	Australia	KU923672	KU923704	KU923710	KU923716	KU923688	-	-	Fu et al. (2019)[1]
C. chrysanthemi	CBS 126518*	*Carthamus* sp.	Netherlands	JQ948271	JQ948601	JQ948932	JQ949592	JQ949922	-	-	Diao et al. (2017)[4]
C. cosmi	CBS 853.73*	*Cosmos* sp, seed	Netherlands	JQ948274	JQ948604	JQ948935	JQ949595	JQ949925	-	-	Fu et al. (2019)[1]
C. costaricense	CBS 330.75*	Coffea arabica	Costa Rica	JQ948180	JQ948510	JQ948841	JQ949501	JQ949831	-	-	Fu et al. (2019)[1]
C. cuscutae	IMI 304802*	*Cuscuta* sp.	Dominica	JQ948195	JQ948525	JQ948856	JQ949516	JQ949846	-	-	Fu et al. (2019)[1]
C. eriobotryae	BCRC FU31138*	*Eriobotrya japonica*	Taiwan	MF772487	MF795423	MN191653	MN191648	MF795428	-	-	Damm et al. (2020)[5]
C. fioriniae	CBS 128517*	*Fiorinia externa*	USA	JQ948292	JQ948622	JQ948953	JQ949613	JQ949943	-	-	Diao et al. (2017)[4]
C. fioriniae	CBS 126526	*Primula* sp., leaf	Netherlands	JQ948323	JQ948653	JQ948984	JQ949644	JQ949974	-	-	Fu et al. (2019)[1]
C. godetiae	CBS 133.44*	*Clarkia hybrida* cv. kelvon	Denmark	JQ948402	JQ948733	JQ949063	JQ949723	JQ950053	-	-	Fu et al. (2019)[1]
C. guajavae	IMI 350839*	*Psidium guajava*	India	JQ948270	JQ948600	JQ948931	JQ949591	JQ949921	-	-	Diao et al. (2017)[4]
C. indonesiense	CBS 127551*	*Eucalyptus* sp.	Indonesia	JQ948288	JQ948618	JQ948949	JQ949609	JQ949939	-	-	Damm et al. (2012)[6]
C. johnstonii	CBS 128532*	*Solanum lycopersicum*, fruit	New Zealand	JQ948444	JQ948775	JQ949105	JQ949765	JQ950095	-	-	Fu et al. (2019)[1]
C. kinghornii	CBS 198.35*	*Phormium* sp.	UK	JQ948454	JQ948785	JQ949115	JQ949775	JQ950105	-	-	Fu et al. (2019)[1]
C. laticiphilum	CBS 112989*	*Hevea brasiliensis*	India	JQ948289	JQ948619	JQ948950	JQ949610	JQ949940	-	-	Fu et al. (2019)[1]
C. limetticola	CBS 114.14*	*Citrus aurantifolia*	USA, Florida	JQ948193	JQ948523	JQ948854	JQ949514	JQ949844	-	-	Damm et al. (2012)[6]
C. lupini	CBS 109225*	*Lupinus albus*	Ukraine	JQ948155	JQ948485	JQ948816	JQ949476	JQ949806	-	-	Fu et al. (2019)[1]
C. melonis	CBS 159.84*	*Cucumis melo*	Brazil	JQ948194	JQ948524	JQ948855	JQ949515	JQ949845	-	-	Damm et al. (2012)[6]
C. nymphaeae	CBS 515.78*	*Nymphaea* sp.	Netherlands	JQ948197	JQ948527	JQ948858	JQ949518	JQ949848	-	-	Diao et al. (2017)[4]
C. nymphaeae	CBS 129929	*Fragaria × ananassa*	USA	JQ948229	JQ948559	JQ948890	JQ949550	JQ949880	-	-	Damm et al. (2012)[6]
C. orchidophilum	CBS 632.80*	*Dendrobium* sp.	USA	JQ948151	JQ948481	JQ948812	JQ949472	JQ949802	-	-	Fu et al. (2019)[1]
C. paranaense	CBS 134729*	*Malus domestica*	Brazil, Parana	KC204992	KC205026	KC205043	KC205077	KC205060	-	-	Braganca et al. (2016)[7]
C. paxtonii	IMI165753*	*Musa* sp.	Saint Lucia	JQ948285	JQ948615	JQ948946	JQ949606	JQ949936	-	-	Damm et al. (2012)[6]

Species	Culture[a]	Host/Tissue	Country/Region	GenBank accession number[b]							Ref.
				ITS	*GAPDH*	*CHS1*	*ACT*	*TUB2*	*CAL*	ApMAT	
C. phormii	CBS 118194*	*Phormium* sp.	Germany	JQ948446	JQ948777	JQ949107	JQ949767	JQ950097	-	-	Fu et al. (2019)[1]
C. pyricola	CBS 128531*	*Pyrus communis*, fruit	New Zealand	JQ948445	JQ948776	JQ949106	JQ949766	JQ950096	-	-	Fu et al. (2019)[1]
C. rhombiforme	CBS 129953*	*Olea europaea*	Portugal	JQ948457	JQ948788	JQ949118	JQ949778	JQ950108	-	-	Fu et al. (2019)[1]
C. salicis	CBS 607.94*	*Salix* sp., leaf.	Netherlands	JQ948460	JQ948791	JQ949121	JQ949781	JQ950111	-	-	Fu et al. (2019)[1]
C. scovillei	CBS 126529*	*Capsicum* sp.	Indonesia	JQ948267	JQ948597	JQ948928	JQ949588	JQ949918	-	-	Diao et al. (2017)[4]
C. simmondsii	CBS 122122*	*Carica papaya*	Australia	JQ948276	JQ948606	JQ948937	JQ949597	JQ949927	-	-	Fu et al. (2019)[1]
C. sloanei	IMI 364297*	*Theobroma cacao*, leaf	Malaysia	JQ948287	JQ948617	JQ948948	JQ949608	JQ949938	-	-	Fu et al. (2019)[1]
C. tamarilloi	CBS 129814*	*Solanum betaceum*, fruit	Colombia	JQ948184	JQ948514	JQ948845	JQ949505	JQ949835	-	-	Fu et al. (2019)[1]
C. walleri	CBS 125472*	*Coffea* sp., leaf	Vietnam	JQ948275	JQ948605	JQ948936	JQ949596	JQ949926	-	-	Fu et al. (2019)[1]
C. wanningense	CGMCC 3.18936*	*Hevea brasiliensis*	China	MG830462	MG830318	MG830302	MG830270	MG830286	-	-	Cao et al. (2019)[8]
C. boninensis species complex											
C. annellatum	CBS 129826*	*Hevea brasiliensis*, leaf	Colombia	JQ005222	JQ005309	JQ005396	JQ005570	JQ005656	JQ005743	-	Fu et al. (2019)[1]
C. beeveri	CBS 128527*	*Brachyglottis repanda*	New Zealand	JQ005171	JQ005258	JQ005345	JQ005519	JQ005605	JQ005692	-	Fu et al. (2019)[1]
C. boninense	CBS 123755*	*Crinum asiaticum* var. sinicum	Japan	JQ005153	JQ005240	JQ005327	JQ005501	JQ005588	JQ005674	-	Fu et al. (2019)[1]
C. boninense	CBS 128506	*Solanum lycopersicum*,fruit	New Zealand	JQ005157	JQ005244	JQ005331	JQ005505	JQ005591	JQ005678	-	Fu et al. (2019)[1]
C. boninense	CBS 128549	*Solanum betaceum*, flowers	New Zealand	JQ005156	JQ005243	JQ005330	JQ005504	JQ005590	JQ005677	-	Damm et al. (2012)[9]
C. boninense	CBS 128526	Dacrycarpus dacrydioides, leaf	New Zealand	JQ005162	JQ005249	JQ005336	JQ005510	JQ005596	JQ005683	-	Douanla-Meli and Unger (2017)[10]
C. brasiliense	CBS 128501*	Passiflora edulis, fruit	Brazil	JQ005235	JQ005322	JQ005409	JQ005583	JQ005669	JQ005756	-	Fu et al. (2019)[1]
C. brassicola	CBS 101059*	*Brassica oleracea* var. gemmifera, leaf	New Zealand	JQ005172	JQ005259	JQ005346	JQ005520	JQ005606	JQ005693	-	Damm et al. (2012)[9]
C. camelliae-japonicae	CGMCC 3.18118*	*Camellia japonica*	Japan	KX853165	KX893584	-	KX893576	KX893580	-	-	Fu et al. (2019)[1]
C. citricola	CBS 134228*	*Citrus unshiu*	China	KC293576	KC293736	KC293792	KC293616	KC293656	KC293696	-	Huang et al. (2013)[11]
C. colombiense	CBS 129818*	*Passiflora edulis*, leaf	Colombia	JQ005174	JQ005261	JQ005348	JQ005522	JQ005608	JQ005695	-	Fu et al. (2019)[1]
C. constrictum	CBS 128504*	*Citrus limon*, fruit	New Zealand	JQ005238	JQ005325	JQ005412	JQ005586	JQ005672	JQ005759	-	Fu et al. (2019)[1]
C. cymbidiicola	IMI 347923*	*Cymbidium* sp., leaf	Australia	JQ005166	JQ005253	JQ005340	JQ005514	JQ005600	JQ005687	-	Fu et al. (2019)[1]
C. dacrycarpi	CBS 130241*	*Dacrycarpus dacrydioides*, leaf	New Zealand	JQ005236	JQ005323	JQ005410	JQ005584	JQ005670	JQ005757	-	Fu et al. (2019)[1]
C. gloeosporioides	IMI 356878*	*Citrus sinensis*	Italy	JX010152	JX010056	JX009818	JX009531	JX010445	JX009731	-	Fu et al. (2019)[1]
C. hippeastri	CBS 125376*	*Hippeastrum vittatum*, leaf	China	JQ005231	JQ005318	JQ005405	JQ005579	JQ005665	JQ005752	-	Fu et al. (2019)[1]
C. karstii	CGMCC 3.14194*	*Vanda sp.*	China	HM585409	HM585391	HM582023	HM581995	HM585428	HM582013		Youlian et al. (2011)[12]
C. karstii	CBS 129833	*Musa* sp.	Mexico	JQ005175	JQ005262	JQ005349	JQ005523	JQ005609	JQ005696	-	Guarnaccia et al. (2017)[2]

Species	Culture[a]	Host/Tissue	Country/Region	GenBank accession number[b]							Ref.
				ITS	*GAPDH*	*CHS1*	*ACT*	*TUB2*	*CAL*	ApMAT	
C. karstii	CBS 129829	*Gossypium hirsutum*	Germany	JQ005189	JQ005276	JQ005363	JQ005537	JQ005623	JQ005710	-	Guarnaccia et al. (2017)[2]
C. karstii	CBS 126532	*Citrus* sp.	South Africa	JQ005209	JQ005296	JQ005383	JQ005557	JQ005643	JQ005730	-	Guarnaccia et al. (2017)[2]
C. karstii	CBS 128550	*Annona cherimola*, fruit	Mexico	JQ005219	JQ005306	JQ005393	JQ005567	JQ005653	JQ005740	-	Damm et al. (2012)[9]
C. karstii	CBS 129824	*Musa* sp., fruit	Colombia	JQ005215	JQ005302	JQ005389	JQ005563	JQ005649	JQ005736	-	Damm et al. (2012)[9]
C. novae-zelandiae	CBS 128505*	Capsicum annuum, fruit	New Zealand	JQ005228	JQ005315	JQ005402	JQ005576	JQ005662	JQ005749	-	Fu et al. (2019)[1]
C. oncidii	CBS 129828*	*Oncidium* sp., leaf	Germany	JQ005169	JQ005256	JQ005343	JQ005517	JQ005603	JQ005690	-	Douanla-Meli and Unger (2017)[10]
C. parsonsiae	CBS 128525	*Parsonsia capsularis*, leaf	New Zealand	JQ005233	JQ005320	JQ005407	JQ005581	JQ005667	JQ005754	-	Fu et al. (2019)[1]
C. petchii	CBS 378.94*	*Dracaena marginata*, leaf	Italy	JQ005223	JQ005310	JQ005397	JQ005571	JQ005657	JQ005744	-	Fu et al. (2019)[1]
C. philodendricola	CGMCC 3.19290*	*Philodendron tatei*	China	MH105257	MH105261	MH105265	MH105273	MH105277	MH105281	-	Xue et al. (2020)[13]
C. phyllanthi	CBS 175.67*	*Phyllanthus acidus*	India	JQ005221	JQ005308	JQ005395	JQ005569	JQ005655	JQ005742	-	Damm et al. (2012)[9]
C. pseudoboninense	CGMCC 3.19755*	*Philodendron. tatei*	China	MK796540	MK796573	-	MK796547	MK796554	-	-	Xue et al. (2020)[13]
C. pseudoboninense	CBS 123921	*Dendrobium kingianum*	Japan	JQ005163	JQ005250	JQ005337	JQ005511	JQ005597	JQ005684	-	Xue et al. (2020)[13]
C. torulosum	CBS 128544*	*Solanum melongena*	New Zealand	JQ005164	JQ005251	JQ005338	JQ005512	JQ005598	JQ005685	-	Fu et al. (2019)[1]
C. gloeosporioides sepcies coomplex											
C. aenigma	ICMP 18608*	*Persea americana*	Israel	JX010244	JX010044	JX009774	JX009443	JX010389	JX009683	KM360143	Vieira et al. (2017)[14]
C. aeschynomenes	ICMP 17673*	*Aeschynomene virginica*	USA	JX010176	JX009930	JX009799	JX009483	JX010392	JX009721	KM360145	Wang et al. (2016)[15]
C. alatae	CBS 304.67*	*Dioscorea alata*	India	JX010190	JX009990	JX009837	JX009471	JX010383	JX009738	KC888932	Wang et al. (2016)[15]
C. alienum	ICMP 12071*	*Malus domestica*	New Zealand	JX010251	JX010028	JX009882	JX009572	JX010411	JX009654	KC888927	Vieira et al. (2017)[14]
C. aotearoa	ICMP 18537*	*Coprosma* sp.	New Zealand	JX010205	JX010005	JX009853	JX009564	JX010420	JX009611	KC888930	Wang et al. (2016)[15]
C. asianum	CBS 130418*	*Coffea arabica*	Thailand	FJ972612	JX010053	JX009867	JX009584	JX010406	FJ917506	FR718814	Vieira et al. (2017)[14]
C. boninense	CBS 123755*	*Crinum asiaticum* var. sinicum	Japan	JQ005153	JQ005240	JQ005327	JQ005501	JQ005588	JQ005674	-	Fu et al. (2019)[1]
C. camelliae	ICMP 18542	*Camellia sasanqua*	USA	JX010223	JX009994	JX009857	JX009488	JX010429	JX009628	KJ954627	Wang et al. (2016)[15]
C. chrysophilum	CMM 4268*,	*Musa* sp.	Brazil	KX094252	KX094183	KX094083	KX093982	KX094285	KX094063	KX094325	Vieira et al. (2017)[14]
C. clidemiae	ICMP 18658*	*Clidemia hirta*	USA	JX010265	JX009989	JX009877	JX009537	JX010438	JX009645	KC888929	Wang et al. (2016)[15]
C. communis	MTCC 11599*	*Mangifera indica*	India	JQ894681	JQ894632	JQ894617	JQ894546	JQ894602	KC790791	JQ894582	Vieira et al. (2017)[14]
C. conoides	CGMCC 3.17615*	*Capsicum annuum*	China	KP890168	KP890162	KP890156	KP890144	KP890174	KP890150	-	Fu et al. (2019)[1]
C. cordylinicola	ICMP 18579*	*Cordyline fruticosa*	Thailand	JX010226	JX009975	JX009864	HM470235	JX010440	HM470238	JQ899274	Wang et al. (2016)[15]
C. dianesei	CMM 4085*	*Mangifera indica*	Brazil	KC329813	KX094158	KX094096	KC533740	KX094270	KX094051	KX094306	Vieira et al. (2017)[14]
C. endomangiferae	CMM 3814*	*Mangifera indica*	Brazil	KC702994	KC702955	KC598113	KC702922	KM404170	KC992372	KJ155453	Vieira et al. (2017)[14]
C. endophytica	CGMCC 3.17887	*Camellia sinensis*	China	KU251561	KU252015	KU251909	KU251642	KU252169	KU251804	KU251734	Wang et al. (2016)[15]
C. fructicola	CBS 130416*	*Coffea arabica*	Thailand	JX010165	JX010033	JX009866	FJ907426	JX010405	FJ917508	JQ807838	Vieira et al. (2017)[14]

Species	Culture[a]	Host/Tissue	Country/Region	GenBank accession number[b]							Ref.
				ITS	*GAPDH*	*CHS1*	*ACT*	*TUB2*	*CAL*	ApMAT	
C. fructicola	CBS 125397	*Tetragastris panamensis*	Panama	JX010173	JX010032	JX009874	JX009581	JX010409	JX009674	JQ807839	Vieira et al. (2017)[14]
C. fructicola	CGMCC3.17889	*Camellia sinensis*	China	KU251520	KU251974	KU251868	KU251601	KU252152	KU251763	KU251706	Wang et al. (2016)[15]
C. gloeosporioides	CBS 112999*	*Citrus sinensis*	Italy	JX010152	JX010056	JX009818	JX009531	JX010445	JX009731	JQ807843	Vieira et al. (2017)[14]
C. grossum	CAUG7*	*Capsicum* sp.	China	KP890165	KP890159	KP890153	KP890141	KP890171	KP890147	-	Diao et al. (2017)[4]
C. henanense	CGMCC 3.17354*	*Camellia sinensis*	China	KJ955109	KJ954810	-	KM023257	KJ955257	KJ954662	KJ954524	Wang et al. (2016)[15]
C. horii	ICMP 10492*	*Diospyros kaki*	Japan	GQ329690	GQ329681	JX009752	JX009438	JX010450	JX009604	JQ807840	Vieira et al. (2017)[14]
C. hymenocallidis	ICMP 18642*	*Hymenocallis americana*	China	JX010278	JX010019	GQ856730	GQ856775	JX010410	JX009709	JQ807842	Vieira et al. (2017)[14]
C. jasmini-sambac	ICMP 19118*	*Jasminum sambac*	Vietnam	HM131511	HM131497	JX009895	HM131507	JX010415	JX009713	JQ807841	Vieira et al. (2017)[14]
C. jiangxiense	CGMCC 3.17363*	*Camellia sinensis*	China	KJ955201	KJ954902	-	KJ954471	KJ955348	KJ954752	KJ954607	Wang et al. (2016)[15]
C. kahawae subsp. ciggaro	ICMP 18539*	*Olea europaea*	Australia	JX010230	JX009966	JX009800	JX009523	JX010434	JX009635	-	Weir et al. (2012)[16]
C. kahawae subsp. kahawae	ICMP 17816*	*Coffea arabica*	Kenya	JX010231	JX010012	JX009813	JX009452	JX010444	JX009642	JQ894579	Weir et al. (2012)[16]
C. ledongense	CGMCC 3.18888*	*Hevea brasiliensis*	China, Hainan	MG242009	MG242017	MG242019	MG242015	MG242011	MG242013	-	Liu et al. (2018)[3]
C. melanocaulon	CBS 133251*	*Vaccinium macrocarpon*	USA	JX145144	KX094187	KX094110	KX093987	KX094290	KX094036	JX145313	Vieira et al. (2017)[14]
C. murrayae	CBS 133239*	*Murraya* sp.	China	JQ247633	JQ247609	-	JQ247657	JQ247644	JQ247596	-	Vieira et al. (2017)[14]
C. musae	CBS 116870*	*Musa* sp.	USA	JX010146	JX010050	JX009896	JX009433	HQ596280	JX009742	KC888926	Vieira et al. (2017)[14]
C. nupharicola	CBS 470.96*	*Nuphar lutea*	USA	JX010187	JX009972	JX009835	JX009437	JX010398	JX009663	JX145319	Wang et al. (2016)[15]
C. proteae	CBS 132882*	*Protea* sp.	South Africa	KC297079	KC297009	KC296986	KC296940	KC297101	KC296960	-	Wang et al. (2016)[15]
C. psidii	CBS 145.29*	*Psidium* sp.	Italy	JX010219	JX009967	JX009901	JX009515	JX010443	JX009743	KC888931	Weir et al. (2012)[16]
C. queenslandicum	ICMP 1778*	*Carica papaya*	Australia	JX010276	JX009934	JX009899	JX009447	JX010414	JX009691	KC888928	Vieira et al. (2017)[14]
C. rhexiae	CBS 133134*	*Rhexia virginica*	USA	JX145128	-	-	-	JX145179	-	JX145290	Wang et al. (2016)[15]
C. salsolae	ICMP 19051*	*Salsola tragus*	Hungary	JX010242	JX009916	JX009863	JX009562	JX010403	JX009696	KC888925	Vieira et al. (2017)[14]
C. siamense	CBS 130417*	*Coffea arabica*	Thailand	JX010171	JX009924	JX009865	FJ907423	JX010404	FJ917505	JQ899289	Vieira et al. (2017)[14]
C. syzygicola	MFLUCC 10-0624*	*Syzygium samarangense*	Thailand	KF242094	KF242156	-	KF157801	KF254880	KF254859	-	Wang et al. (2016)[15]
C. temperatum	CBS 133122*	*Vaccinium macrocarpon*	USA	JX145159	-	-	-	JX145211	-	JX145298	Wang et al. (2016)[15]
C. theobromicola	CBS 124945*	*Theobroma cacao*	Panama	JX010294	JX010006	JX009869	JX009444	JX010447	JX009591	KC790726	Vieira et al. (2017)[14]
C. ti	ICMP 4832*	*Cordyline* sp.	New Zealand	JX010269	JX009952	JX009898	JX009520	JX010442	JX009649	KM360146	Wang et al. (2016)[15]
C. tropicale	CBS 124949*	*Theobroma cacao*	Panama	JX010264	JX010007	JX009870	JX009489	GU994454	JX009719	GU994425	Vieira et al. (2017)[14]
C. viniferum	GZAAS 5.08601*	*Vitis vinifera*	China	JN412804	JN412798	-	JN412795	JN412813	JQ309639	-	Vieira et al. (2017)[14]
C. wuxiense	CGMCC 3.17894*	*Camellia sinensis*	China	KU251591	KU252045	KU251939	KU251672	KU252200	KU251833	KU251722	Wang et al. (2016)[15]
C. xanthorrhoeae	CBS 127831*	*Xanthorrhoea preissii*	Australia	JX010261	JX009927	JX009823	JX009478	JX010448	JX009653	KC790689	Weir et al. (2012)[16]

[a]ICMP, International Collection of Microorganisms from Plants (New Zealand); CBS, Centraalbureau voor Schimmelcultures (Netherlands); COAD: Coleção Octávio Almeida Drummond, Viçosa, Brazil; CGMCC, China General Microbiological Culture Collection Center (China); MFLUCC: Mae Fah Luang University Culture Collection, Chiang Rai, Thailand; CMM: Culture Collection of Phytopathogenic Fungi, Prof. Maria Menezes, Federal Rural University of Pernambuco, Brazil; GZAAS, Guizhou Academy of Agricultural Sciences (China); BCRC, Bioresource Collection and Research Center, Hsinchu, Taiwan; *indicates the ex-type cultures.
[b]ITS: internal transcribed spacer; *GAPDH*: glyceraldehyde 3-phosphate dehydrogenase; *CHS-1*: chitin synthase; *ACT*: actin; *TUB2*: beta-tubulin; *CAL*: calmodulin; ApMAT: intergenic sequence between *Apn2* DNA lyase and *MAT1-2-1*.
[c]-: not available.

References

1 Fu, M. *et al.* *Colletotrichum* species associated with anthracnose of *Pyrus* spp. in China. *Persoonia* **42**, 1-35 (2019).

2 Guarnaccia, V., Groenewald, J. Z., Polizzi, G. & Crous, P. W. High species diversity in *Colletotrichum* associated with citrus diseases in Europe. *Persoonia* **39**, 32-50 (2017).

3 Liu, X. *et al.* *Colletotrichum* species causing anthracnose of rubber trees in China. *Sci. Rep.* **8**, 10435 (2018).

4 Diao, Y. Z. *et al.* *Colletotrichum* species causing anthracnose disease of chili in China. *Persoonia* **38**, 20-37 (2017).

5 Damm, U., Sun, Y. C. & Huang, C. J. *Colletotrichum eriobotryae* sp. nov. and *C. nymphaeae*, the anthracnose pathogens of loquat fruit in central Taiwan, and their sensitivity to azoxystrobin. Mycological Progress 19, 367-380 (2020).

6 Damm, U., Cannon, P. F., Woudenberg, J. H. C. & Crous, P. W. The *Colletotrichum acutatum* species complex. *Stud. Mycol.*, 37-113 (2012).

7 Braganca, C. A. D., Damm, U., Baroncelli, R., Massola, N. S. & Crous, P. W. Species of the *Colletotrichum acutatum* complex associated with anthracnose diseases of fruit in Brazil. *Fungal Biol.* **120**, 547-561 (2016).

8 Cao, X. R., Xu, X. M., Che, H. Y., West, J. S. & Luo, D. Q. Three *Colletotrichum* species, including a new species, are associated to leaf anthracnose of rubber tree in Hainan, China. *Plant Dis.* **103**, 117-124 (2019).

9 Damm, U. *et al.* The *Colletotrichum boninense* species complex. *Stud. Mycol.*, 1-36 (2012).

10 Douanla-Meli, C. & Unger, J. G. Phylogenetic study of the *Colletotrichum* species on imported citrus fruits uncovers a low diversity and a new species in the *Colletotrichum gigasporum* complex. *Fungal Biol* **121**, 858-868 (2017).

11 Huang, F. *et al.* *Colletotrichum* species associated with cultivated citrus in China. *Fungal Divers.* **61**, 61-74 (2013).

12 Youlian, Y., Cai, L., Yu, Z., Liu, Z. & Hyde, K. D. *Colletotrichum* species on *Orchidaceae* in southwest China. *Cryptogamie Mycol.* **32**, 229-253, 225 (2011).

13 Xue, L. *et al.* Characterization and Pathogenicity of *Colletotrichum* Species on *Philodendron tatei* cv. Congo in Gansu Province, China. *Plant Dis.* **0**, null (2020).

14 Vieira, W. A. S. *et al.* The impact of phenotypic and molecular data on the inference of *Colletotrichum* diversity associated with *Musa*. *Mycologia* **109**, 912-934 (2017).

15 Wang, Y. C. *et al.* Diverse *Colletotrichum* species cause anthracnose of tea plants (*Camellia sinensis* (L.) O. Kuntze) in China. *Sci. Rep.* **6** (2016).
16 Weir, B. S., Johnston, P. R. & Damm, U. The *Colletotrichum gloeosporioides* species complex. *Stud. Mycol.*, 115-180 (2012).

Supplementary Table S2. Conidia and appressoria measurements of five *Colletotrichum* spp. isolated in this study and type strains

		Conidia			Appressoria			
Species	Accession No.[a]	Length x Width (μm)[b]	L/W ratio	Medium	Length x Width (μm)[b]	L/W ratio	Medium	Ref.
C. acutatum species complex								
C. miaoliense sp. nov.	This study	14.2 ± 1.1 × 4.1 ± 0.3	3.5	1/4 PDA	7.5 ± 1.1 × 5.0 ± 0.6	1.5	dH_2O[c]	This study
C. acutatum	CBS 112996*	12.6 ± 1.8 × 3.9 ± 0.3	3.2	SNA	7.3 ± 2.0 × 5.4 ± 1.2	1.3	SNA	Damm et al. (2012)[1]
C. *nymphaeae*	CBS 515.78*	16.1 ± 2.3 × 4.9 ± 0.7	3.3	SNA	8.7 ± 2.5 × 5.5 ± 1.0	1.6	SNA	Damm et al. (2012)[1]
C. simmondsii	CBS 122122*	8.1 ± 1.7 × 2.9 ± 0.4	2.7	SNA	7.8 ± 1.9 × 5.3 ± 1.1	1.5	SNA	Damm et al. (2012)[1]
C. boninensis species complex								
C. boninense	This study	15.2 ± 1.1 × 6.8 ± 0.5	2.3	PDA	8.9 ± 1.3 × 6.1 ± 0.7	1.5	dH_2O[c]	This study
C. boninense	CBS 123755*	12.8 ± 1.6 × 5.4 ± 0.4	2.4	SNA	10.5 ± 3.3 × 6.4 ± 1.5	1.6	SNA	Damm et al. (2012)[2]
C. karstii	This study	15.5 ± 1.2 × 6.5 ± 0.4	2.4	1/4 PDA	8.7 ± 1.2 × 6.4 ± 0.8	1.4	dH_2O[c]	This study
C. karstii	CGMCC3.14194*	15.4 ± 1.3 × 6.5 ± 0.5	2.4	PDA	9.3 ± 1.6 × 6.4 ± 0.9	1.5	PDA	Youlian et al. (2011)[3]
C. gloeosporioides sepcies coomplex								
C. fructicola	This study	13.0 ± 0.7 × 5.3 ± 0.4	2.5	1/4 PDA	8.1 ± 0.9 × 6.3 ± 1.0	1.3	dH_2O[c]	This study
C. fructicola	CBS 130416*	11.5 ± 1.0 × 3.6 ± 0.3	3.2	PDA	7.4 ± 1.3 × 4.5 ± 0.9	1.6	PDA	Prihastuti et al. (2009)[4]; Weir et al. (2012)[5]
C. siamense	This study	14.5 ± 0.9 × 4.9 ± 0.3	3.0	1/4 PDA	8.4 ± 0.9 × 5.7 ± 0.5	1.5	dH_2O[c]	This study
C. siamense	CBS 130417*	10.2 ± 1.7 × 3.5 ± 0.4	2.9	PDA	6.7 ± 1.1 × 4.1 ± 0.4	1.6	PDA	Prihastuti et al. (2009)[4]; Weir et al. (2012)[5]

[a]* indicates type strains.

[b]Data are mean ± standard deviation.

[c]Appressoria were induced in dH_2O on a microscopic slide.

References

1 Damm, U., Cannon, P. F., Woudenberg, J. H. C. & Crous, P. W. The *Colletotrichum acutatum* species complex. *Stud. Mycol.*, 37-113 (2012).

2 Damm, U. *et al.* The *Colletotrichum boninense* species complex. *Stud. Mycol.*, 1-36 (2012).

3 Youlian, Y., Cai, L., Yu, Z., Liu, Z. & Hyde, K. D. *Colletotrichum* species on *Orchidaceae* in southwest China. *Cryptogamie Mycol.* **32**, 229-253, 225 (2011).

4 Prihastuti, H., Cai, L., Chen, H., McKenzie, E. H. C. & Hyde, K. D. Characterization of *Colletotrichum* species associated with coffee berries in northern Thailand. *Fungal Divers.* **39**, 89-109 (2009).

5 Weir, B. S., Johnston, P. R. & Damm, U. The *Colletotrichum gloeosporioides* species complex. *Stud. Mycol.*, 115-180 (2012).

Supplementary Table S3. Mycelial growth rates (mm day^{-1}) of *Colletotrichum* spp. under different temperatures

Temperature (°C)	*C. siamense* ML133	*C. fructicola* ML348	*C. karstii* ML351	*C. boninense* ML521	*C. miaoliense* ML1040
18	6.5 ± 0.1 a	6.1 ± 0.0 b	5.6 ± 0.1 c	6.0 ± 0.0 b	3.9 ± 0.0 d
22	6.5 ± 0.2 a	7.2 ± 0.4 a	5.3 ± 0.2 b	7.2 ± 0.1 a	4.5 ± 0.2 b
25	9.1 ± 1.0 a	9.4 ± 0.6 a	8.2 ± 0.3 a	9.1 ± 0.3 a	6.0 ± 0.2 b
28	10.3 ± 0.6 a	8.7 ± 0.2 b	7.4 ± 0.4 b	8.4 ± 0.2 b	5.7 ± 0.3 c
30	10.0 ± 0.3 a	7.7 ± 0.2 b	7.5 ± 0.3 b	6.4 ± 0.6 bc	5.6 ± 0.4 c
32	7.5 ± 0.8 a	5.6 ± 0.2 b	5.8 ± 0.3 b	1.5 ± 0.2 d	3.6 ± 0.4 c

The results from the same temperature were analyzed together. Data (mean ± standard error) with different letters are significantly different based on Tukey's range test at $P < 0.05$.

Supplementary Table S4. Mycelial growth rates (mm day^{-1}) of *Colletotrichum* spp. at 25°C and 30°C

Temp. (°C)	*C. siamense*			*C. fructicola*		*C. karstii*		*C. miaoliense*	
	ML133	ML540	ML612	ML356	ML368	ML442	ML1792	ML1042	ML1794
25	11.9 ± 0.2 a	12.0 ± 0.2 a	11.4 ± 0.3 ab	10.6 ± 0.2 bcd	10.7 ± 0.2 bc	9.7 ± 0.2 d	9.7 ± 0.2 cd	7.7 ± 0.1 e	7.5 ± 0.1 e
30	11.8 ± 0.2 a	12.0 ± 0.3 a	10.0 ± 0.6 bc	8.6 ± 0.2 cd	10.5 ± 0.2 b	8.3 ± 0.3 d	8.9 ± 0.3 cd	7.0 ± 0.2 e	6.6 ± 0.2 e

The results from the same temperature were analyzed together. Data (mean ± standard error) with different letters are significantly different based on Tukey's range test at $P < 0.05$.

Supplementary Table S5. Primers used in this study

Gene/Locus	Primer name	Sequence	Reference
Internal transcribed spacer (ITS)	ITS1	5'- TCC GTA GGT GAA CCT GCG G -3'	White et al (1990)[1]
	ITS4	5'- TCC TCC GCT TAT TGA TAT GC -3'	White et al (1990)[1]
Glyceraldehyde-3-phosphate dehydrogenase (*GAPDH*)	GDF3	5'- GCC GTC AAC GAC CCC TTC ATT GA -3'	This study
	GDR3	5'-TTC TCG TTG ACA CCC ATC ACG TAC ATG -3'	This study
Chitin synthase (*CHS-1*)	CHS-79F	5'- TGG GGC AAG GAT GCT TGG AAG AAG -3'	Carbone and Kohn (1999)[2]
	CHS-345R	5'- TGG AAG AAC CAT CTG TGA GAG TTG -3'	Carbone and Kohn (1999)[2]
Actin (*ACT*)	ACT-512F	5'- ATG TGC AAG GCC GGT TTC GC -3'	Carbone and Kohn (1999)[2]
	ACT-783R	5'- TAC GAG TCC TTC TGG CCC AT -3'	Carbone and Kohn (1999)[2]
β-Tubulin 2 (*TUB2*)	T1	5'- AAC ATG CGT GAG ATT GTA AGT -3'	O'Donnell and Cigelnik (1997)[3]
	T2	5'- TAG TGA CCC TTG GCC CAGT TG -3'	O'Donnell and Cigelnik (1997)[3]
Calmodulin (*CAL*)	CL1C	5'- GAA TTC AAG GAG GCC TTC TC -3'	Weir et al (2012)[4]
	CL2C	5'- CTT CTG CAT CAT GAG CTG GAC -3'	Weir et al (2012)[4]
Intergenic sequence between *Apn2* DNA lyase and *Mat1-2-1* (ApMAT)	APF-long	5'- TCA TTC TAC GTA TGT GCC CGC CCG TTG -3'	This study; Silva et al. (2012)[5]
	APR-long	5'- CCA GAA ATA CAC CGA ACT TGC AAA GAT -3'	This study; Silva et al. (2012)[5]

References

1 White, T. J., Bruns, T., Lee, S. & Taylor, J. in *PCR protocols: a guide to methods and applications* Vol. 18 (eds M.A. Innis, D.H. Gelfand, J.J. Sninsky, & T.J. White) 315-322 (Academic Press, Inc., 1990).

2 Carbone, I. & Kohn, L. M. A method for designing primer sets for speciation studies in filamentous ascomycetes. *Mycologia* **91**, 553-556 (1999).

3 O'Donnell, K. & Cigelnik, E. Two divergent intragenomic rDNA ITS2 types within a monophyletic lineage of the fungus *Fusarium* are nonorthologous. *Mol Phylogenet Evol* **7**, 103-116 (1997).

4 Weir, B. S., Johnston, P. R. & Damm, U. The *Colletotrichum gloeosporioides* species complex. *Stud. Mycol.*, 115-180 (2012).

5 Silva, D. N. *et al.* Application of the *Apn2*/*MAT* locus to improve the systematics of the *Colletotrichum gloeosporioides* complex: an example from coffee (*Coffea* spp.) hosts. *Mycologia* **104**, 396-409 (2012).

Supplementary Table S6. Nucleotide substitution models used in phylogenetic analyses

Gene/Locus[1]	Acutatum clade[2]	Boninenses clade[2]	Gloeosporioides clade[2]
ITS	HKY+I	SYM+I+G	GTR+I
GAPDH	HKY+G	HKY+I	HKY+I
CHS-1	K80+I	K80+G	K80+G
ACT	GTR+G	GTR+G	HKY+G
TUB2	GTR+G	HKY+I	GTR+G
CAL	-	GTR+G	GTR+G
ApMAT	-	-	HKY+G

[1]ITS: internal transcribed spacer; *GAPDH*: glyceraldehyde 3-phosphate dehydrogenase; *CHS-1*: chitin synthase; *ACT*: actin; *TUB2*: beta-tubulin; *CAL*: calmodulin; ApMAT: intergenic sequence between *Apn2* DNA lyase and *MAT1-2-1*. [2]K80**:** Kimura 2-parameter; HKY: Hasegawa-Kishino-Yano; SYM: symmetrical model; GTR: general time reversible; G: gamma distribution; I: proportion of invariable sites.
